# MDM2 Antagonist Idasanutlin Reduces HDAC1/2 Abundance and Corepressor Partners but Not HDAC3

**DOI:** 10.1021/acsmedchemlett.3c00449

**Published:** 2023-12-06

**Authors:** Joshua P. Smalley, Shaun M. Cowley, James T. Hodgkinson

**Affiliations:** † Leicester Institute of Structural and Chemical Biology, School of Chemistry, University of Leicester, Leicester LE1 7RH, United Kingdom; ‡ Department of Molecular and Cell Biology, University of Leicester, Leicester LE1 7RH, United Kingdom

**Keywords:** Histone Deacetylase, HDAC, MDM2, P53, Proteolysis Targeting Chimera, PROTAC, Idasanutlin

## Abstract

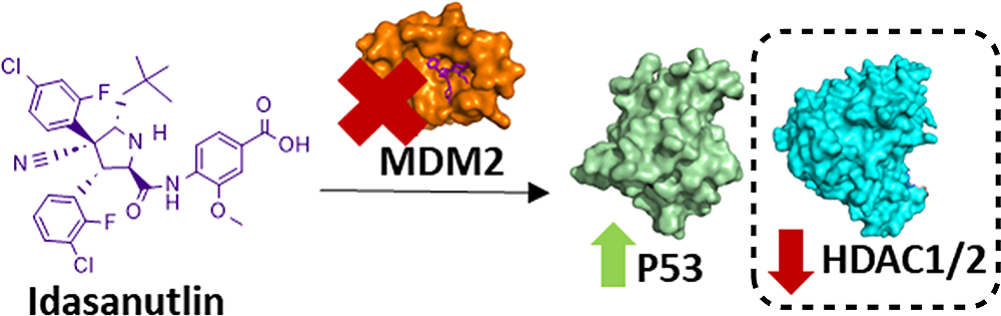

Histone deacetylases 1–3 (HDAC1, HDAC2, and HDAC3) and their associated corepressor complexes play important roles in regulating chromatin structure and gene transcription. HDAC enzymes are also validated drug targets for oncology and offer promise toward new drugs for neurodegenerative diseases and cardiovascular diseases. We synthesized four novel heterobifunctional molecules designed to recruit the mouse double minute 2 homologue (MDM2) E3 ligase to degrade HDAC1–3 utilizing the MDM2 inhibitor idasanutlin, known as proteolysis targeting chimeras (PROTACs). Idasanutlin inhibits the MDM2-P53 protein–protein interaction and is in clinical trials. Although two MDM2-recruiting heterobifunctional molecules reduced HDAC1 and HDAC2 abundance with complete selectivity over HDAC3 and reduced HDAC1/2 corepressor components LSD1 and SIN3A, we were surprised to observe that idasanutlin alone was also capable of this effect. This finding suggests an association between the MDM2 E3 ligase and HDAC1/2 corepressor complexes, which could be important for designing future dual/bifunctional HDAC- and MDM2-targeting therapeutics, such as PROTACs.

Histone deacetylase enzymes (HDACs) are validated drug targets, and FDA-approved HDAC inhibitors are used to treat hematological cancers.^1^ Of the 18 HDAC enzymes present in humans, 11 are zinc-dependent, and the remaining seven are NAD^+^ dependent.^2^ Of the class I HDAC enzymes, HDAC1, HDAC2, and HDAC3 exist in the nucleus as catalytic deacetylase subunits of large multiprotein corepressor complexes.^3^ These HDACs and their associated corepressor complexes play an important role in modifying chromatin structure and gene transcription.^4^ The selective targeting of HDAC1/2 and HDAC3 and their associated corepressor complexes has received significant attention as a potential strategy to harness the therapeutic benefits of HDAC-targeting drugs while reducing the debilitating side effects associated with current FDA-approved pan-HDAC inhibitors.^1,4−7^

Proteolysis targeting chimeras (PROTACs) offer an alternative strategy to drugging proteins of interest and have been receiving copious interest for multiple drug targets.^8^ PROTACs are heterobifunctional molecules that consist of a ligand for the protein of interest, an E3 ligase ligand, and a linker that covalently bonds these two components. PROTACs engage the protein of interest and recruit a E3 ligase to target the protein of interest for polyubiquitination and proteasome-mediated degradation.^9^ We previously reported von Hippel–Lindau (VHL) E3 ligase-recruiting PROTACs that exhibit HDAC1/2 and HDAC3 degradation, such as JPS004 (Figure 1).^10−12^ We also discovered that minor modifications to the VHL E3 ligand can result in the selective degradation of HDAC3 over HDAC1/2 with JPS036.^11^ Other researchers have also reported selective degraders of HDAC3 utilizing VHL and cereblon-recruiting E3 ligase ligands, including PROTACs HD-TAC7 and XZ9002.^13−15^

**Figure 1:**
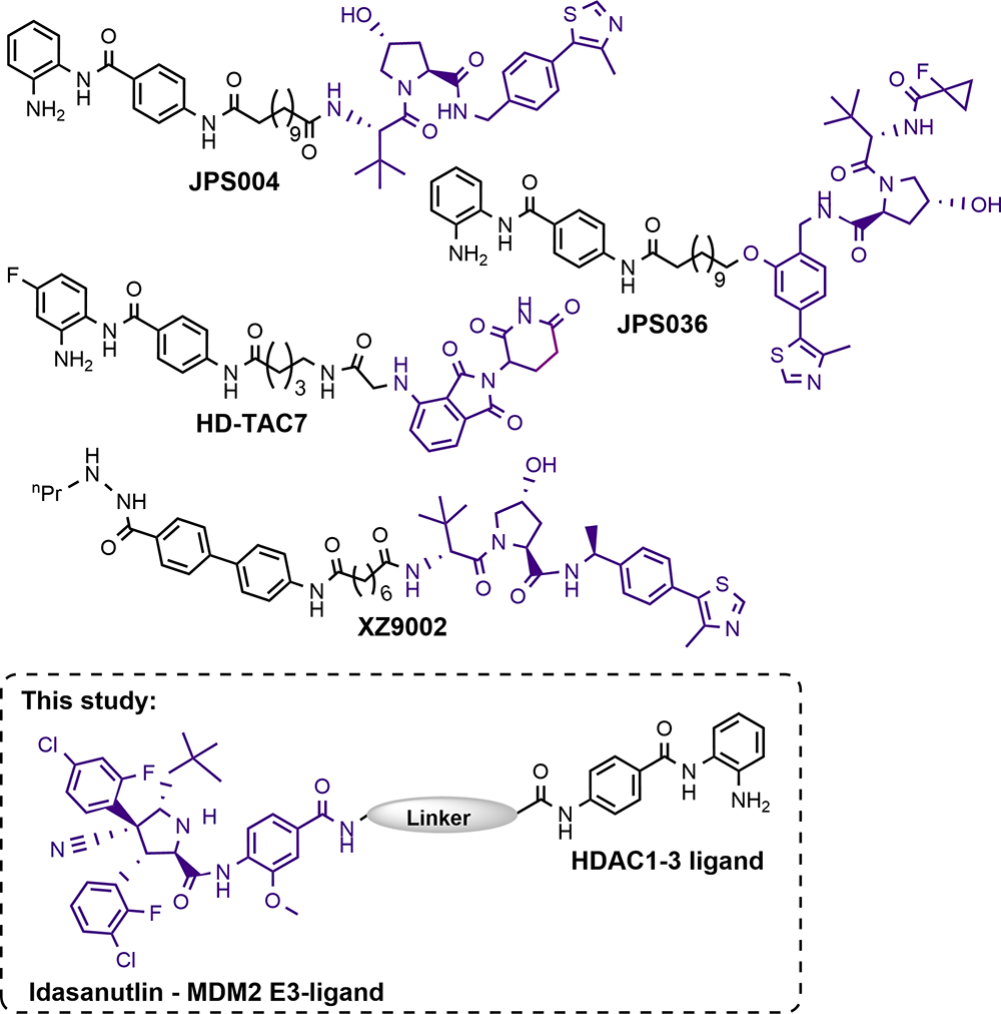
JPS004 is a HDAC1, HDAC2, and HDAC3 degrader.^10,11^ JPS036 degrades HDAC3 with selectivity over HDAC1 and HDAC2.^11^ HD-TAC7 and XZ9002 are also selective degraders of HDAC3.^13,14^ This study involved investigating the incorporation of idasanutlin, an MDM2 E3 ligand, into heterobifunctional molecules in combination with a HDAC1–HDAC3 ligand. E3 ligands are highlighted in purple.

In this study, we set out to investigate HDAC1–3-targeting PROTACs that could recruit the mouse double minute 2 (MDM2) E3 ligase. The incorporation of differing E3 ligands into PROTACs can have profound effects on the protein degradation selectivity observed and degradation potency.^16^ Idasanutlin is a verified MDM2 binder that was chosen as the E3 ligand for this study.^17^ Idasanutlin inhibits the MDM2-P53 protein–protein interaction and is currently in clinical trials.^18^ Inhibition of the MDM2-P53 interaction prevents the MDM2 E3 ligase-initiating proteasome-mediated degradation of the tumor suppressor P53 and induces apoptosis in cancer cells.^19,20^ Idasanutlin has been incorporated into other MDM2-recruiting PROTACs targeting BRD4 for degradation;^21^ however, as far as we are aware, this is the first study that this E3 ligand has been incorporated into PROTACs designed to target HDACs for degradation.

We initially synthesized PROTACs **1** and **2** with alkyl linkers because we had previously found such alkyl-based linkers (12 atoms) to be more effective degraders in VHL E3 ligase-recruiting PROTACs (Scheme 1).^11^ However, we quickly discovered that the hydrophobicity of idasanutlin in combination with the alkyl linkers resulted in compounds with high cLogP values and poor aqueous solubility (cLogP values of 8.62 and 8.48 for **1** and **2**, respectively). In an attempt to overcome poor water solubility, we decided to investigate the PEG-functionalized linkers **3** and **4**, which reduced the cLogP values compared with **1** and **2** (cLogP values of 6.95 and 7.11 for **3** and **4**, respectively). For full physiochemical property predictions of **1**–**4**, see the Supporting Information. These values are not outside the boundaries of PROTACs reported in the literature, and **3** and **4** exhibited enhanced water solubility compared with **1** and **2**.^22^

**Scheme 1:**
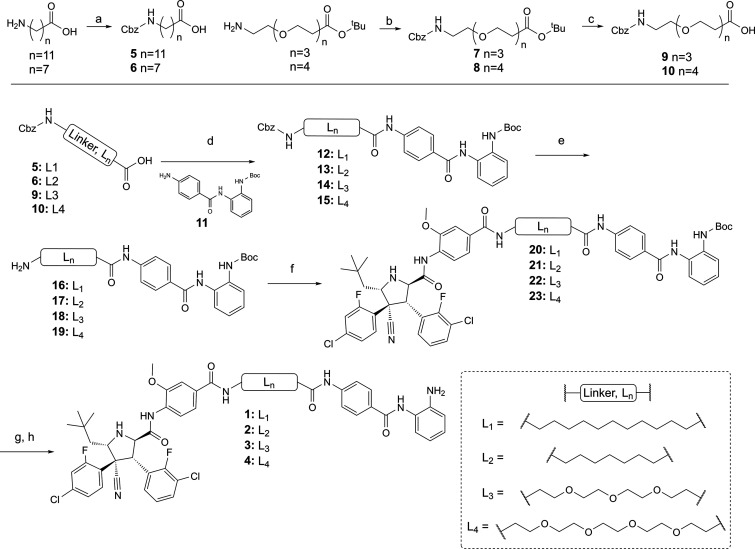
Reagents and Conditions: (a) CbzCl, K_2_CO_3_, THF, rt, overnight, 50–52%; (b) CbzCl, NaHCO_3_ (aq), THF, rt, 2 h, 62–88%; (c) TFA, DCM, rt, 4 h, 99%; (d) HATU, DIPEA, DMF, rt, overnight, 80–86%; (e) H_2_, 10% Pd/C, MeOH, rt, overnight, 95–100%; (f) Idasanutlin, HATU, DIPEA, DMF, rt, overnight, 70–86%; (g) TFA, DCM, rt, 3 h; (h) MP-carbonate resin, MeOH, rt, 2 h, 82–100%

We tested **1**–**4** side-by-side with CI-994, an HDAC1–3 inhibitor, and JPS004, an HDAC1–3 degrader. We recently discovered VHL-based degraders, such as JPS004, exhibit a hook effect for HDAC3 at concentrations greater than 1 μM resulting in compromised HDAC3 degradation at higher concentrations.^11^ Compounds **1**–**4** were screened in HCT116 cells at 10, 1, and 0.1 μM, and HDAC1, HDAC2, and HDAC3 abundance was examined by quantitative Western blotting and compared with DMSO, with CI-994 and JPS004 treated at 10 μM. We were pleased to discover that **3** and **4** reduced HDAC1 and HDAC2 abundance at 10 and 1 μM, with the longer PEG linker **4** exhibiting greater degradation than **3**, which incorporates a shorter linker by one PEG unit (Figure 2A). However, we were even more surprised to discover that **3** and **4** did not reduce HDAC3 abundance at any of the three concentrations tested, thereby suggesting **3** and **4** may act selectively on HDAC1 and HDAC2. We screened **1**–**4** for their effects on histone H3 lysine K56 acetylation (H3K56ac), as previously with JPS004 and CI-994 we observed significant increases in H3K56ac levels compared with DMSO-treated cells alone (Figure 2B). CI-994 and JPS004 increased H3K56ac levels, as previously reported; however, we were surprised to observe that **3** and **4** did not increase H3K56ac levels at all the concentrations tested compared with DMSO controls, despite the clear effects of **3** and **4** on HDAC1 and HDAC2 abundance.

**Figure 2:**
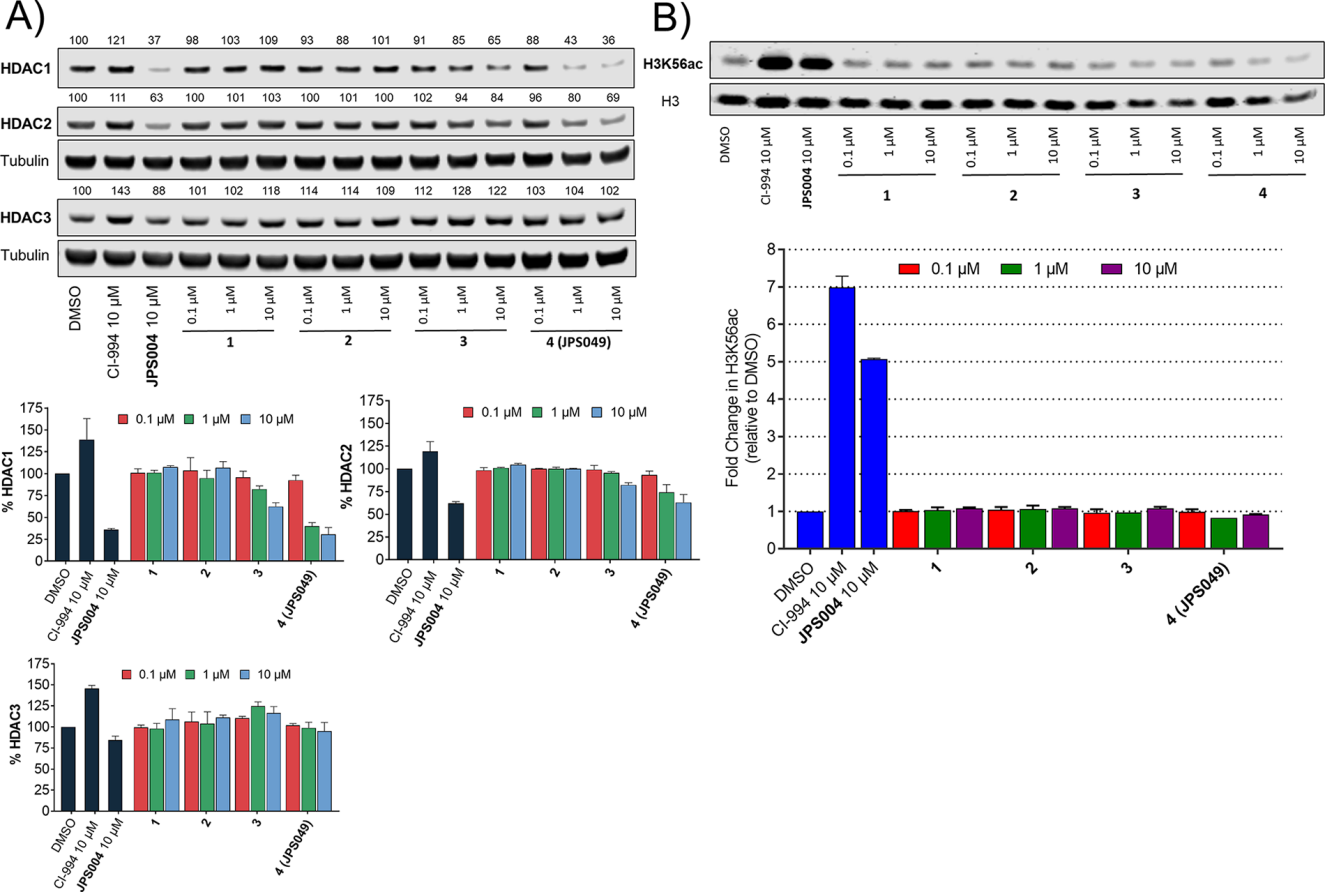
(A) Compounds **1**–**4** were screened at 0.1, 1.0, and 10 μM in HCT116 cells for 24 h and quantified for HDAC1, HDAC2, and HDAC3 abundance by quantitative Western blotting with specific antibodies for HDAC1, HDAC2, and HDAC3. Error bars are representative of *n* = 2 replicates. (B) Compounds **1**–**4** were also quantified for effects on H3K56 acetylation under the same conditions utilizing a specific antibody for H3K56ac.

As compound **4** seemed the most effective HDAC1/2 degrader, we performed dose–response curves with **4** and blotted for HDAC1, HDAC2, and HDAC3 abundance (Figure 3A). Dose-dependent degradation of HDAC1 and HDAC2 was observed with **4**, but again, no degradation of HDAC3 was observed in the presence of **4** at all the concentrations tested. HDAC1 and HDAC2 exist *in vivo* as subunits of six corepressor complexes inducing CoREST, SIN3, MIER, RERE, MiDAC, and NuRD;^3^ we wanted to investigate the effects of **4** on such corepressor complexes. We chose SIN3A and LSD1 as exemplary corepressor components of the Sin3 and CoREST complexes. Initially, we were excited to observe that SIN3A and LSD1 also exhibited a dose-dependent reduction in the presence of **4**, which also correlated very well with the HDAC1 and HDAC2 degradation dose–response curves (Figure 3B). In fact, interestingly, LSD1 dose-dependent reduction was near identical to that observed for HDAC2 dose-dependent reduction.

**Figure 3:**
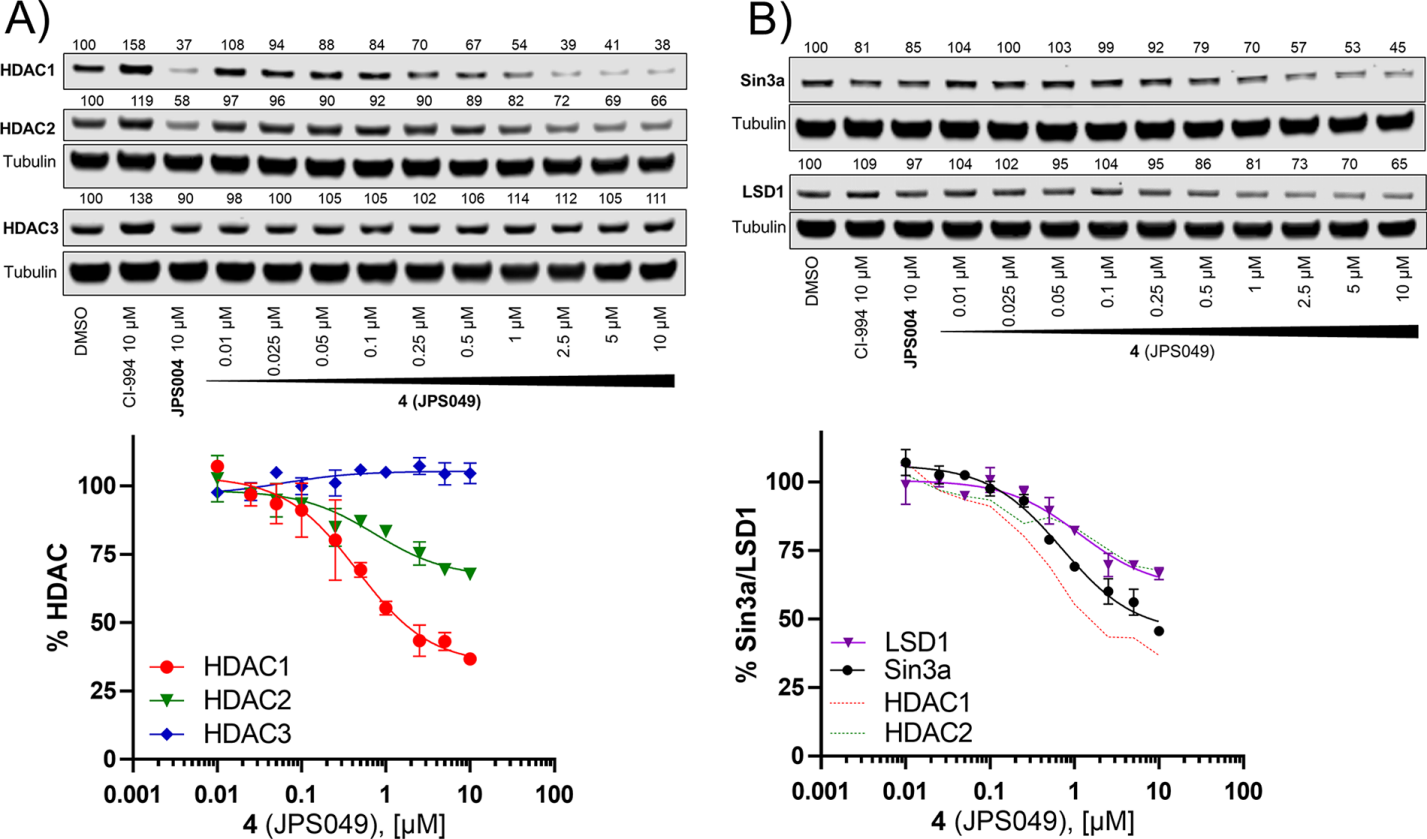
(A) Dose–response curve with **4** and HDAC1, HDAC2, and HDAC3 abundance determined by quantitative Western blotting with specific antibodies for HDAC1, HDAC2, and HDAC3. (B) Dose–response curve with **4** and LSD1 and Sin3a abundance determined by quantitative Western blotting with specific antibodies for Sin3a and LSD1. Error bars are representative of *n* = 2 replicates.

Given **4** did not increase H3K56ac levels similar to Cl-994 and JPS004, we wanted to rule out that the effects we observed on HDAC1 and HDAC2 abundance were not due to the idasanutlin ligand itself incorporated into **4**. Idasanutlin was incubated with HCT116 cells in an identical manner to **4**, and HDAC1, HDAC2 and HDAC3 abundance were quantified as previously performed (Figure 4). To our disappointment, idasanutlin was as effective as **4** in reducing HDAC1 and HDAC2 abundance at 1 and 10 μM with no effect on HDAC3 abundance, as previously observed. This means that **4** unlikely acts as a PROTAC initiating the selective degradation of HDAC1 and HDAC2 by inducing a ternary complex between HDAC1/2 and the E3 ligase MDM2 to promote degradation. Instead, it seems idasanutlin, alone, is capable of reducing HDAC1 and HDAC2 abundance. However, this finding is still interesting in its own right; the complete selectivity for HDAC1 and HDAC2 and the associated corepressor components LSD1 and SIN3A over HDAC3 is noteworthy. Idasanutlin is currently in clinical trials, and we are not aware of other studies that have demonstrated that idasanutlin reduces HDAC1 and HDAC2 abundance and also effects HDAC1/2 corepressor complexes. The mode of action of idasanutlin involves blocking MDM2-initiated P53 degradation via the proteasome, and prevention of this degradation increases P53 levels.^19^ P53, itself, is also subject to post-translational modifications, including acetylation and deacetylation, and contains up to 13 acetylated lysine residues.^23,24^ HDAC2 has been identified to be involved in the deacetylation of lysine 320 (K320ac) in P53 in HCT116 cells.^24,25^ Increased acetylation of P53 has been reported to increase P53 protein stability by Ito et al.:^26^ the authors speculate that P53 acetylation prevents lysine ubiquitination and proteasome-mediated degradation of P53. Intriguingly, Ito et al. reported that the deacetylation of P53 can be mediated by a HDAC1–MDM2 complex, and this complex promotes P53 degradation.^27^ Wagner et al. highlighted that there are a number of E3 ligases that interconnect HDAC2 protein stability with P53, including RNF12, MULE, and the E2 ligase UBCH8, the latter of which can be induced by HDAC inhibition.^24^ On the basis of our findings and those of others, increased P53 levels by idasanutlin may trigger an unidentified E3 ligase (or network of E3 ligases) to reduce HDAC1 and HDAC2 abundance, thereby preventing P53 deacetylation and further stabilizing and enhancing P53 levels. Regarding **4**, we hypothesize that **4** is reaching MDM2 in the nucleus, but perhaps not enough of **4** is also engaging HDAC1 and HDAC2 in the nucleus as a bifunctional PROTAC. This may be due to the fact that idasanutlin is a more potent MDM2 inhibitor (IC_50_ = 6 nM)^19^ than the benzamide HDAC1–3 ligand in **4** (CI-994, HDAC1-CoREST IC_50_ = ∼0.5 μM).^10^ Further fine-tuning of the physiochemical properties of **4** or modifications of the HDAC or MDM2 ligand binding affinities may yet yield PROTACs that recruit MDM2 to degrade HDAC1 and HDAC2, possibly with selectivity for HDAC1/2 over HDAC3. Our study also reveals that there may be beneficial synergistic effects observed with MDM2-recruiting HDAC1/2 PROTACs with the P53 regulation pathway.

**Figure 4:**
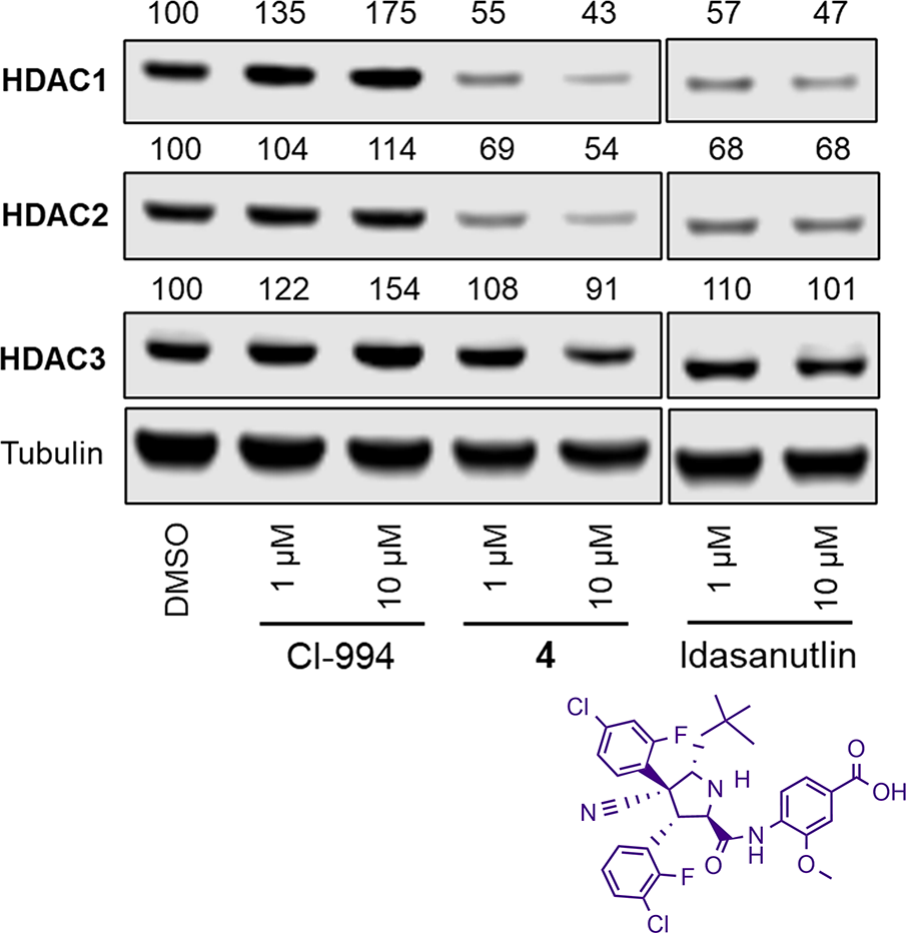
Western blots comparing HDAC1, HDAC2, and HDAC3 abundance in the presence of **4** and idasanutlin at 1 and 10 μM.

## Experimental Procedures

### General Chemical Methods

All reagents were purchased from commercially available sources and used without further purification. Idasanutlin was purchased from MedChemExpress. Preparative column chromatography and flash column chromatography using a Biotage Isolera purification system were both performed by using silica gel 60 (230–400 mesh). Semipreparative HPLC was performed on a ThermoFisher Ultimate 3000 system with Chromeleon software on a Phenomenex Luna C18 column. The mobile phases were water and acetonitrile with a flow rate of 10 mL/min, 45 min gradient. NMR spectra were acquired using a Bruker 400 (^1^H, 400 MHz; ^13^C 101 MHz) instrument at ambient temperature using deuterated solvent as reference. High-resolution mass spectra (HRMS) were recorded on a Water Aquity XEVO Q ToF machine and measured in *m*/*z*. Analytical UPLC-MS were collected on a Xevo G2-XS QToF mass spectrometer (Waters) coupled to an Acquity LC system (Waters) using an Acquity UPLC BEH C18 column (130 Å, 1.7 μm, 2.1 × 50 mm, Waters). The mobile phases were water and acetonitrile with a flow rate of 0.6 mL/min, 10 min gradient. The purities of all final compounds were over 95%, as determined by LC-MS analysis monitored at 260 and 310 nm. HPLC traces for **1**–**4** are included in the Supporting Information. All intermediates and final compounds were fully assigned by ^1^H and ^13^C NMR using 2D NMR spectra (see the Supporting Information for full analysis). No unexpected or unusually high safety hazards were encountered.

### Cell Lines and Cell Culture

HCT116 human colon carcinoma cells were grown in Dulbecco’s modified Eagle medium (GIBCO, 41965-039) supplemented with 10% fetal bovine serum (Sigma) and 1× glutamine/penicillin/streptomycin (GIBCO, 10378-016). This cell line was incubated at 37 °C in 5% CO_2_. Cells were treated with PROTACs (0.01–10 μM) alongside HDACi CI-994 (10 μM).

### Western Blotting

HCT116 cells were seeded into six-well plates (4 × 10^5^ cells/well for 24 h, 2 × 10^5^ cells/well for 48 h) for 24 h and then treated with DMSO or compounds at the indicated concentrations in fresh medium (5 mL total). After treatment, the cells were harvested and lysed in lysis buffer (50 mM Tris-HCl, 150 mM NaCl, 0.5% NP-40, and 0.5% TritonX-100) supplemented with a protease inhibitor (Sigma, P8340). The suspension was incubated on ice for 30 min and centrifuged (18 000 rcf, 15 min, 4 °C); then, the supernatant was collected, and protein concentrations were quantified via Bradford assay using protein assay dye reagent concentrate (BIO-RAD). For histone extraction, an equal volume of 0.4 N H_2_SO_4_ was added to the pellets, and the extracts were placed at 4 °C overnight and centrifuged (18 000 rcf, 15 min, 4 °C); then, the supernatant (histone extract) was collected. Western blots were run on NuPAGE 4–12% bis-Tris gels with 30 μg of protein or 10 μL of acid-extracted histone loaded per lane using NuPAGE LDS sample buffer (4×). PageRuler Plus Prestained Ladder was used for the size standards. After gel electrophoresis at 140 V for 90 min, the separated proteins were transferred to a nitrocellulose membrane at 30 V for 60 min. The membranes were probed with primary antibodies (see the Supporting Information) for 60–90 min. Blots were developed with complementary IRDye-conjugated secondary antibodies, and the bands were visualized using an Odyssey infrared imaging system. Image processing and band intensity quantification were performed by using Image Studio Lite.

## Supporting Information Available

Experimental protocols, ^1^H and ^13^C spectra, mass spectra data, UPLC traces, and Western blots (PDF)

## Abbreviations

HDAC1: histone deacetylase 1
HDAC2: histone deacetylase 2
HDAC3: histone deacetylase 3
MDM2: mouse double minute 2
PROTAC: proteolysis-targeting chimera
P53: tumor protein 53
LSD1: lysine-specific demethylase 1
Sin3: switch-independent protein 3
NAD: nicotinamide adenine dinucleotide
BRD4: bromodomain-containing protein 4
VHL: von Hippel–Lindau
PEG: polyethylene glycol
CoREST: corepressor of repressor element-1 silencing transcription factor
H3K56: histone 3 lysine 56
RNF12: ring finger protein 12
MULE: Mcl-1 ubiquitin ligase E3
UbcH8: ubiquitin-carrier protein H8
