# Supplementary material for: MDM2 Antagonist Idasanutlin Reduces HDAC1/2 Abundance and Corepressor Partners but Not HDAC3
Source: ACS Med Chem Lett. 2023 Dec 6;15(1):93–8. doi: 10.1021/acsmedchemlett.3c00449 (PMC10788946; doi:10.1021/acsmedchemlett.3c00449)
Supplement: Supplementary file 1 — ml3c00449_si_001.pdf [file ml3c00449_si_001.pdf]

Supporting Information

The MDM2 antagonist Idasanutlin reduces HDAC1/2 abundance and corepressor partners but not HDAC3

Joshua P. Smalley,[a] *Shaun M. Cowley*[b*] *and James T. Hodgkinson*[a*]

a. Leicester Institute of Structural and Chemical Biology, School of Chemistry, University of Leicester, Leicester, LE1 7RH, UK

b. Department of Molecular and Cell Biology, University of Leicester, Leicester, LE1 7RH, UK

Table of Contents

1. Synthetic protocols and characterization data 3
2. Properties Table of **1-4** 20
Table S1. Physiochemical properties[a] table for compounds **1-4**. 20
3. Western Blots 21
Figure S1. Screening of 1-4 for HDAC1-HDAC3 degradation. 21
Figure S2. Screening of 1-4 for H3K56ac. 22
Figure S3. HDAC1, HDAC2 and HDAC3 dose response with compound 4. 22
Figure S4. Sin3A and LSD1 dose response with compound 4 23
Figure S5. Idasanutlin effects on HDAC1, HDAC2 and HDAC3. 23
4. NMR spectra for **1-4** 24
5. UPLC traces for **1-4** 28
Figure S6. UPLC results for **1** (JPS331), **2** (JPS333), **3** (JPS334) and **4** (JPS340) at 260nm. 28

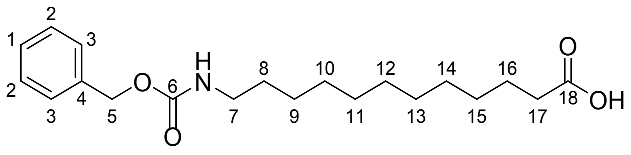

12-(((benzyloxy)carbonyl)amino)dodecanoic acid (5): A mixture of 12-aminododecanoic acid (1.00 g, 4.64 mmol) and K_2CO_3 (1.28 g, 9.28 mmol) were suspended in THF (20 ml), then benzyl chloroformate (0.99 ml, 6.96 mmol) was added and the resulting mixture was stirred at room temperature overnight. The reaction mixture was diluted with EtOAc (15 ml) and water (20 ml), then separated. The aqueous phase acidified to pH 2 with HCl 2M, extracted with EtOAc (2 x 30 mL), then the combined organic phases dried over Na_2SO_4, filtered and evaporated *in vacuo* to afford **5** (0.81 g, 2.32 mmol, 50% yield) as a white solid. ^{1}H NMR (400 MHz, DMSO-d_6) δ_H ppm 11.94 (br s, 1 H, 18-CO_2H), 7.27 - 7.39 (m, 5 H, (1-3)-CH), 7.21 (t, *J*=5.5 Hz, 1 H, 6-NH), 4.99 (s, 2 H, 5-CH_2), 2.95 - 3.01 (m, 2 H, 7-CH_2), 2.18 (t, *J*=7.4 Hz, 2 H, 17-CH_2) 1.44 - 1.51 (m, 2 H, 16-CH_2), 1.34 - 1.39 (m, 2 H, 8-CH_2), 1.22 -1.25 (m, 14 H, (9-15)-CH_2). ^{13}C NMR (101 MHz, DMSO-d_6) δ_C ppm 175.0 (C18), 172.3 (C6), 137.8 (C4), 128.8 (C2), 128.2 (C1), 128.15 (C3), 65.5 (C5), 40.7 (C7), 34.1 (C17), 29.9 (C8), 29.5 (alkyl CH_2), 29.45 (alkyl CH_2), 29.4 (alkyl CH_2), 29.35 (alkyl CH_2), 29.3 (alkyl CH_2), 29.2 (alkyl CH_2), 26.7 (C9), 25.0 (C16). HRMS (ESI) m/z: $[M+H]^+$ calculated for $C_{20}H_{32}NO_4$: 350.2331, 350.2339.

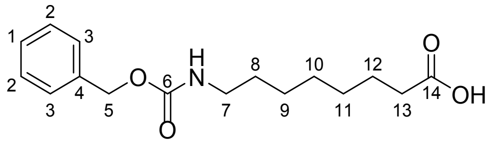

8-(((benzyloxy)carbonyl)amino)octanoic acid (6): A mixture of 8-aminooctanoic acid (0.500 g, 3.14 mmol) and K_2CO_3 (0.868 g, 6.28 mmol) were suspended in THF (15 ml), then benzyl chloroformate (0.67 ml, 4.71 mmol) was added and the resulting mixture was stirred at room temperature overnight. The reaction mixture was diluted with EtOAc (10 ml) and water (20 ml), then separated. The aqueous phase acidified to pH 2 with HCl 2M, extracted with EtOAc (2 x 30 mL), then the combined organic phases dried over Na_2SO_4, filtered and evaporated *in vacuo* to afford **6** (0.478 g, 3.14 mmol, 52% yield) as a white solid. ^{1}H NMR (400 MHz, $CDCl_3$) δ_H ppm 11.29 (br s, 1 H, 14-CO_2H), 7.28 - 7.40 (m, 5 H, (1-3)-CH), 5.10 (s, 2 H, 5-CH_2), 4.78 (br t, *J*=6.5 Hz, 1 H, 6-NH), 3.19 (q, *J*=6.5 Hz, 2 H, 7-CH_2), 2.35 (t, *J*=7.5 Hz, 2 H, 13-CH_2), 1.63 (quin, *J*=6.8 Hz, 2 H, 12-CH_2), 1.46 - 1.54 (m, 2 H, 8-CH_2), 1.28 - 1.37 (m, 6 H, (9-11)-

CH_2). ^{13}C NMR (101 MHz, $CDCl_3$) δ_C ppm 179.3 (C14), 156.4 (C6), 136.6 (C4), 128.5 (C2), 128.1 (C1), 128.05 (C3), 66.6 (C5), 41.0 (C7), 33.9 (C13), 29.8 (C8), 28.9 (C10/11), 28.8 (C10/11), 26.5 (C9), 24.5 (C12). HRMS (ESI) m/z: $[M+Na]^+$ calculated for $C_{16}H_{23}NO_4Na$: 316.1525, found 316.1527.

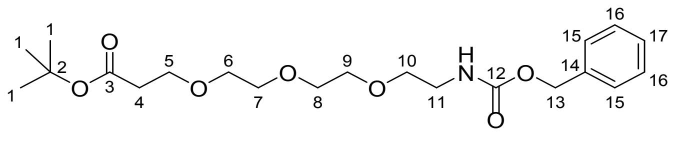

Tert-butyl 3-oxo-1-phenyl-2,7,10,13-tetraoxa-4-azahexadecan-16-oate (7): To a solution of tert-butyl 3-(2-(2-(2-aminoethoxy)ethoxy)ethoxy)propanoate (0.410 g, 1.48 mmol) in THF (8 mL), benzyl chloroformate (0.317 mL, 2.22 mmol) and sat. $NaHCO_3$ (2 mL) were added, then the resultant solution stirred at room temperature for 2 hours. The reaction mixture was diluted with water (5 mL), extracted with EtOAc (3 x 15 mL), then the combined organic extracts washed with water (20 mL) and sat. NaCl (20 mL), dried over $MgSO_4$, filtered and concentrated *in vacuo* to afford a colourless oil (0.719 g). The crude product was purified by column chromatography (10-100% EtOAc in hexane) to afford **7** (0.379 g, 0.92 mmol, 62% yield) as a colourless oil. ^1H NMR (400 MHz, $CDCl_3$) δ_H ppm 7.28 - 7.37 (m, 5 H, (15-17)-CH), 5.36 (br s, 1 H, 12-NH), 5.10 (s, 2 H, 13-CH_2), 3.68 (t, *J*=6.5 Hz, 2 H, 5-CH_2), 3.58 - 3.63 (m, 8 H, (6-9)-CH_2), 3.56 (t, *J*=5.1 Hz, 2 H, 10-CH), 3.39 (q, *J*=5.2 Hz, 2 H, 11-CH_2), 2.48 (t, *J*=6.5 Hz, 2 H, 4-CH_2), 1.44 (s, 9 H, 1-CH_3). ^{13}C NMR (101 MHz, $CDCl_3$) δ_C ppm 170.8 (C3), 156.4 (C12), 136.6 (C14), 128.4 (C16), 128.1 (C17), 128.0 (C15), 80.4 (C2), 70.5 (2 x alkoxy CH_2), 70.3 (alkoxy CH_2), 70.3 (alkoxy CH_2), 70.0 (C10), 66.8 (C5), 66.6 (C13), 40.8 (C11), 36.2 (C4), 28.0 (C1). HRMS (ESI) m/z: $[M+H]^+$ calculated for $C_{21}H_{34}NO_7$: 412.2335, found 412.2336.

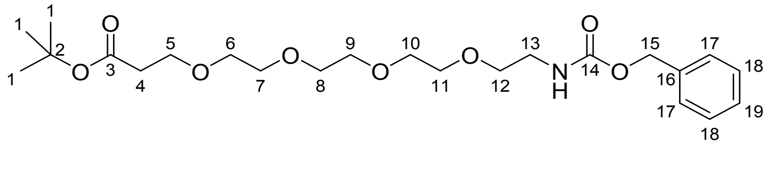

Tert-butyl 3-oxo-1-phenyl-2,7,10,13,16-pentaoxa-4-azanonadecan-19-oate (8): To a solution of tert-Butyl 1-amino-3,6,9,12-tetraoxapentadecan-15-oate (0.250 g, 0.778 mmol) in THF (8 mL), benzyl chloroformate (0.167 mL, 1.17 mmol) and sat. $NaHCO_3$ (2 mL) were added, then the resultant solution stirred at room temperature for 2 hours. The reaction mixture was diluted with water (5 mL), extracted with EtOAc (3 x 15 mL), then the combined organic extracts washed with water (20 mL) and sat. NaCl (20 mL), dried over $MgSO_4$, filtered and

concentrated *in vacuo* to afford a colourless oil (0.513 g). The crude product was purified by column chromatography (10-100% EtOAc in hexane) to afford **8** (0.314 g, 0.689 mmol, 88% yield) as a colourless oil. ^{1}H NMR (400 MHz, $CDCl_3$) δ_H ppm 7.28 - 7.39 (m, 5 H, (17-19)-CH), 5.36 (t, J=5.2 Hz, 1 H, 13-NH), 5.10 (s, 2 H, 15-CH_2), 3.69 (t, *J*=6.6 Hz, 2 H, 5-CH_2), 3.61 - 3.66 (m, 8 H, (8,9,10,11)-CH_2), 3.53 - 3.61 (m, 6 H, (6,7,12)-CH_2), 3.39 (q, *J*=5.2 Hz, 2 H, 13-CH_2), 2.49 (t, *J*=6.6 Hz, 2 H, 4-CH_2), 1.45 (s, 9 H, 1-CH_3). ^{13}C NMR (101 MHz, $CDCl_3$) δ_C ppm 170.8 (C3), 156.4 (C14), 136.6 (C16), 128.4 (C18), 128.1 (C19), 128.0 (C17), 80.5 (C2), 70.6 (alkoxy CH_2), 70.5 (alkoxy CH_2), 70.5 (alkoxy CH_2), 70.4 (alkoxy CH_2), 70.3 (alkoxy CH_2), 70.3 (alkoxy CH_2), 70.0 (C12), 66.8 (C5), 66.6 (C15), 40.9 (C5), 36.2 (C4), 28.1 (C1). HRMS (ESI) m/z: $[M+H]^+$ calculated for $C_{23}H_{37}NO_8Na$: 478.2417, found 478.2414.

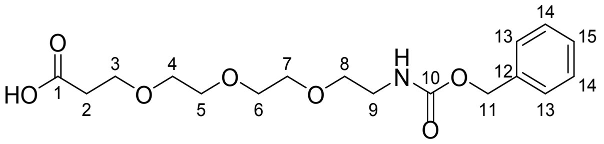

3-oxo-1-phenyl-2,7,10,13-tetraoxa-4-azahexadecan-16-oic acid (9): TFA (2 mL) was added to a stirring solution of **7** (0.345 g, 0.838 mmol) in DCM (4 mL) and the resulting reaction mixture stirred at room temperature for 4 hours. The reaction mixture was concentrated *in vacuo* to afford **9** (0.296 g, 0.831 mmol, 99% yield) as a pale yellow tar. ^{1}H NMR (400 MHz, CD_3CN) δ_H ppm 7.29 - 7.41 (m, 5 H, (13-15)-CH), 5.75 (br s, 1 H, 10-NH), 5.05 (s, 2 H, 11-CH_2), 3.65 (t, *J*=6.2 Hz, 2 H, 3-CH_2), 3.54 (d, *J*=1.3 Hz, 8 H, (4-7)-CH_2), 3.48 (t, *J*=5.6 Hz, 2 H, 8-CH_2), 3.26 (q, *J*=5.6 Hz, 2 H, 9-CH_2), 2.50 (t, *J*=6.2 Hz, 2 H, 2-CH_2). CO_2H not visible. ^{13}C NMR (101 MHz, CD_3CN) δ_C ppm 173.4 (C1), 157.6 (C2), 138.5 (C12), 129.5 (C14), 128.9 (C15), 128.8 (C13), 71.2 (C4/5/6/7), 71.1 (C4/5/6/7), 71.05 (C4/5/6/7), 71.0 (C4/5/6/7), 70.5 (C8), 67.3 (C3), 66.9 (C11), 41.6 (C2), 35.4 (C9). HRMS (ESI) m/z: $[M+H]^+$ calculated for $C_{17}H_{26}NO_7$: 356.1709, found 356.1699.

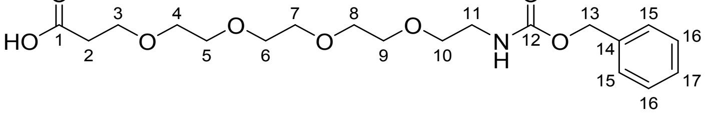

3-oxo-1-phenyl-2,7,10,13-tetraoxa-4-azahexadecan-16-oic acid (10): TFA (2 mL) was added to a stirring solution of **10** (0.314 mg, 0.688 mmol) in DCM (2 mL) and the resulting reaction mixture stirred at room temperature for 4 hours. The reaction mixture was concentrated *in vacuo* to afford **10** (0.275 g, 0.681 mmol, 99% yield) as a clear colourless oil.

^1H NMR (400 MHz, CD_3CN) δ_H ppm 7.27 - 7.41 (m, 5 H, (15-17)-CH), 5.77 (t, *J*=5.6 Hz, 1 H, 11-NH), 5.05 (s, 2 H, 13-CH_2), 3.66 (t, *J*=6.2 Hz, 2 H, 3-CH_2), 3.51 - 3.56 (m, 12 H, (4-9)-CH_2), 3.49 (t, *J*=5.5 Hz, 2 H, 10-CH_2), 3.26 (q, *J*=5.6 Hz, 2 H, 11-CH_2), 2.50 (t, *J*=6.2 Hz, 2 H, 2-CH_2). CO_2H not visible. ^{13}C NMR (101 MHz, CD_3CN) δ_C ppm 173.4 (C1), 157.6 (C12), 138.5 (C14), 129.5 (C16), 128.9 (C17), 128.8 (C15), 71.25 (alkoxy CH_2), 71.2 (alkoxy CH_2), 71.15 (alkoxy CH_2), 71.1 (alkoxy CH_2), 71.05 (alkoxy CH_2), 71.0 (alkoxy CH_2), 70.5 (C10), 67.3 (C3), 67.0 (C13), 41.6 (C11), 35.4 (C2). HRMS (ESI) m/z: $[M+H]^+$ calculated for $C_{19}H_{30}NO_8$: 400.1971, found 400.1973.

General method A for the synthesis of 12-15

To a solution of carboxylic acid linker intermediate (1.1-1.3 equiv.) in dry DMF (10 mL/mmol) at 0 °C, DIPEA (3 equiv.) and HATU (1.3-1.5 equiv.) were added. The reaction mixture was stirred for 15 minutes, after which a solution of amine HDACi (1 equiv.) in DMF was added slowly and the resultant solution stirred at room temperature overnight. The reaction mixture was diluted in EtOAc, then washed with sat. $NaHCO_3$ and sat. NaCl. The organic layer was dried over $MgSO_4$, filtered and concentrated *in vacuo* to give the corresponding crude, which was chromatographically purified to afford the desired compound.

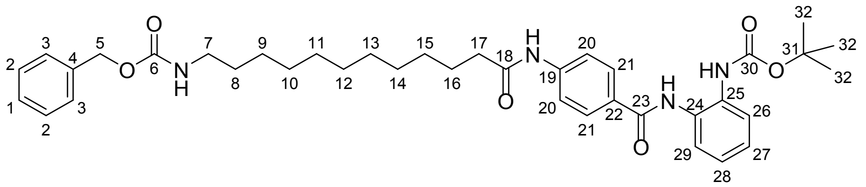

Tert-butyl (2-(4-(12-(((benzyloxy)carbonyl)amino)dodecanamido)benzamido)-phenyl)carbamate (12): Following general method A, **12** was obtained from **5** (192 mg, 0.550 mmol) and **11** (150 mg, 0.458 mmol). The crude product was purified by column chromatography (10-80% EtOAc in hexane) to afford **12** (250 mg, 0.379 mmol, 83% yield) as an off-white solid. ^1H NMR (400 MHz, DMSO-d_6) δ_H ppm 10.16 (s, 1 H, 18-NH), 9.74 (s, 1 H, 23-NH), 8.67 (br s, 1 H, 30-NH), 7.91 (d, *J*=8.8 Hz, 2 H, 21-CH), 7.74 (d, *J*=8.8 Hz, 2 H, 20-CH), 7.48 - 7.57 (m, 2 H, (26,29)-CH), 7.27 - 7.39 (m, 5 H, (1-3)-CH), 7.09 - 7.24 (m, 3 H, (27,28)-CH,6-NH), 5.00 (s, 2 H, 5-CH_2), 2.97 (q, *J*=6.7 Hz, 2 H, 7-CH_2), 2.34 (t, *J*=7.4 Hz, 2 H, 17-CH_2), 1.60 (quin, *J*=6.9 Hz, 2 H, 16-CH_2), 1.45 (s, 9 H, 32-CH_3), 1.35 - 1.41 (m, 2 H, 8-CH_2), 1.21 - 1.32 (m, 14 H, (9-15)-CH_2). ^{13}C NMR (101 MHz, DMSO-d_6) δ_C ppm 171.8 (C18), 164.7 (C23), 156.1 (C6), 153.5 (C28), 142.6 (C19), 137.3 (C4), 131.6 (C25), 130.0 (C24),

128.5 (C21), 128.3 (C2), 128.2 (C22), 127.7 (C1), 127.65 (C3), 125.9 (C29), 125.4 (C28), 124.2 (C27), 123.9 (C26), 118.2 (C20), 79.7 (C31), 65.0 (C5), 40.2 (C7), 36.5 (C17), 29.4 (C8), 29.0 (alkyl CH_2), 28.95 (alkyl CH_2), 28.9 (alkyl CH_2), 28.8 (alkyl CH_2), 28.7 (alkyl CH_2), 28.65 (alkyl CH_2), 28.0 (C32), 26.2 (C9), 25.0 (C16). HRMS (ESI) m/z: $[M+H]^+$ calculated for $C_{38}H_{51}N_4O_6$: 659.3809, found 659.3776.

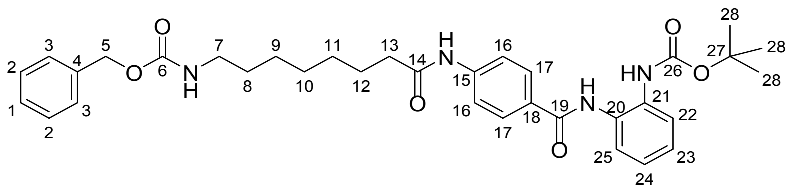

Tert-butyl (2-(4-(8-(((benzyloxy)carbonyl)amino)octanamido)benzamido)phenyl)-carbamate (13): Following general method A, **13** was obtained from **6** (215 mg, 0.733 mmol) and **11** (200 mg, 0.611 mmol). The crude product was purified by column chromatography (0-100% EtOAc in hexane) to afford **13** (317 mg, 0.526 mmol, 86% yield) as an off-white solid. ^{1}H NMR (400 MHz, DMSO-d_6) δ_H ppm 10.17 (s, 1 H, 14-NH), 9.74 (s, 1 H, 19-NH), 8.67 (br s, 1 H, 26-NH), 7.91 (d, J=8.7 Hz, 2 H, 17-CH), 7.74 (d, J=8.7 Hz, 2 H, 16-CH), 7.50 - 7.56 (m, 2 H, (22,25)-CH), 7.27 - 7.38 (m, 5 H, (1-3)-CH), 7.12 - 7.24 (m, 3 H, (23,24)-CH,6-NH), 5.00 (s, 2 H, 5-CH_2), 2.98 (q, J=6.7 Hz, 2 H, 7-CH_2), 2.34 (t, J=7.4 Hz, 2 H, 13-CH_2), 1.60 (quin, J=6.7 Hz, 2 H, 12-CH_2), 1.45 (s, 9 H, 28-CH_3), 1.37 - 1.43 (m, 2 H, 8-CH_2), 1.23 - 1.32 (m, 6 H, (9-11)-CH_2). ^{13}C NMR (101 MHz, DMSO-d_6) δ_C ppm 171.8 (C14), 164.7 (C19), 156.1 (C6), 153.5 (C24), 142.5 (C15), 137.3 (C4), 131.6 (C21), 130.0 (C20), 128.5 (C17), 128.3 (C2), 128.2 (C18), 127.7 (C1), 127.65 (C3), 125.9 (C25), 125.4 (C24), 124.2 (C23), 123.9 (C22), 118.2 (C16), 79.7 (C27), 65.0 (C5), 40.2 (C7), 36.5 (C13), 29.4 (C8), 28.6 (C11), 28.5 (C10), 28.0 (C28), 26.1 (C9), 24.9 (C12). HRMS (ESI) m/z: $[M+H]^+$ calculated for $C_{34}H_{43}N_4O_6$: 603.3183, found 603.3187.

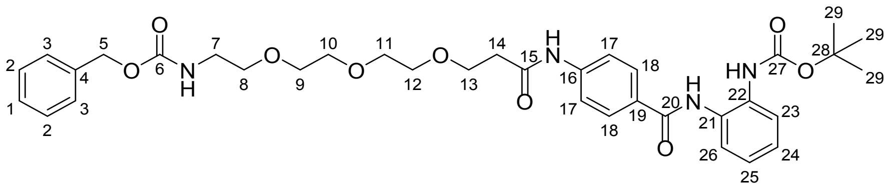

Tert-butyl (2-(4-(3-oxo-1-phenyl-2,7,10,13-tetraoxa-4-azahexadecan-16-amido)-benzamido)phenyl)carbamate (14): Following general method A, **14** was obtained from **9**

(261 mg, 0.734 mmol) and **11** (200 mg, 0.611 mmol). The crude product was purified by column chromatography (10-100% EtOAc in hexane) to afford **14** (335 mg, 0.499 mmol, 82% yield) as a pale yellow solid. ^{1}H NMR (400 MHz, $CDCl_3$) δ_H ppm 9.17 (br s, 1 H, 21-NH), 8.88 (br s, 1 H, 16-NH), 7.89 (d, *J*=8.7 Hz, 2 H, 18-CH), 7.63 - 7.74 (m, 3 H, (17,26)-CH), 7.28 - 7.38 (m, 6 H, (1,3,5,23)-CH), 7.13 - 7.20 (m, 2 H, (24,25)-CH), 7.10 (s, 1 H, 22-NH), 5.34 (t, *J*=5.5 Hz, 1 H, 7-NH), 5.05 (s, 2 H, 5-CH_2), 3.77 (t, *J*=5.4 Hz, 2 H, 13-CH_2), 3.65 - 3.70 (m, 4 H, (11,12)-CH_2), 3.57 - 3.64 (m, 4 H, (9,10)-CH_2), 3.51 (t, *J*=5.2 Hz, 2 H, 8-CH_2), 3.34 (q, *J*=5.5 Hz, 2 H, 7-CH_2), 2.62 (t, *J*=5.4 Hz, 2 H, 14-CH_2), 1.50 (s, 9 H, 29-CH_3). ^{13}C NMR (101 MHz, $CDCl_3$) δ_C ppm 170.3 (C15), 165.3 (C20), 156.6 (C6), 154.5 (C27), 141.6 (C16), 136.4 (C4), 130.6 (C21), 130.4 (C22), 129.2 (C19), 128.5 (C2), 128.4 (C18), 128.1 (C3), 128.0 (C1), 125.9 (C24/25), 125.7 (C26), 125.6 (C24/25), 124.4 (C23), 119.3 (C17), 81.1 (C28), 70.4 (alkoxy CH_2), 70.2 (alkoxy CH_2), 70.1 (alkoxy CH_2), 70.1 (alkoxy CH_2), 70.1 (alkoxy CH_2), 66.8 (C13), 66.7 (C5), 40.8 (C7), 37.7 (C14), 28.3 (C29). HRMS (ESI) m/z: $[M+H]^+$ calculated for $C_{35}H_{45}N_4O_9$: 665.3187, found 665.3196.

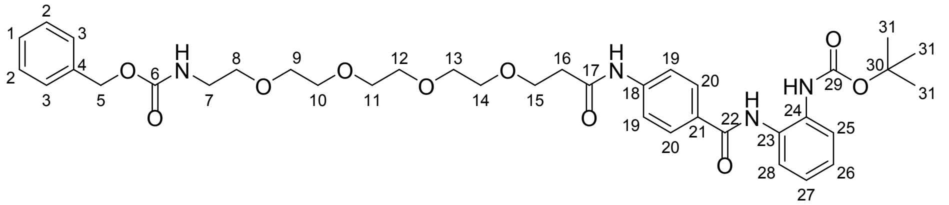

Tert-butyl (2-(4-(3-oxo-1-phenyl-2,7,10,13,16-pentaoxa-4-azanonadecan-19-amido)benzamido)phenyl)carbamate (15) Following general method A, **15** was obtained from **10** (285 mg, 0.713 mmol) and **11** (195 mg, 0.596 mmol). The crude product was purified by column chromatography (10-100% EtOAc in hexane) to afford **15** (338 mg, 0.477 mmol, 80% yield) as a pale yellow solid. ^{1}H NMR (400 MHz, $CDCl_3$) δ_H ppm 9.15 (br s, 1 H, 23-NH), 8.99 (br s, 1 H, 16-NH), 7.91 (d, *J*=8.6 Hz, 2 H, 20-CH), 7.65 - 7.75 (m, 3 H, (19,28)-CH), 7.29 - 7.37 (m, 6 H, (1,2,3,25)-CH), 7.11 - 7.22 (m, 2 H, (26,27)-CH), 7.03 (s, 1 H, 24-NH), 5.45 (t, *J*=5.4 Hz, 1 H, 7-NH), 5.08 (s, 2 H, 5-CH_2), 3.78 (t, *J*=5.4 Hz, 2 H, 15-CH_2), 3.60 - 3.68 (m, 8 H, (11,12,13,14)-CH_2), 3.54 - 3.58 (m, 4 H, (9,10)-CH_2), 3.49 - 3.53 (m, 2 H, 8-CH_2), 3.34 (q, *J*=5.4 Hz, 2 H, 7-CH_2), 2.63 (t, *J*=5.4 Hz, 2 H, 16-CH_2), 1.51 (s, 9 H, 31-CH_2). ^{13}C NMR (101 MHz, $CDCl_3$) δ_C ppm 170.3 (C17), 165.2 (C22), 156.5 (C6), 154.5 (C29), 141.8 (C18), 136.5 (C4), 130.8 (C23), 130.3 (C24), 129.1 (C21), 128.5 (C2), 128.4 (C20), 128.1 (C3), 128.0 (C1), 125.9 (C26/27), 125.7 (C26/27,C28), 124.4 (C25), 119.3 (C19), 81.2 (C30), 70.5 (2 x alkoxy CH_2), 70.45 (alkoxy CH_2), 70.2 (2 x alkoxy CH_2), 70.1 (alkoxy CH_2), 70.0 (alkoxy

CH_2), 66.9 (C15), 66.6 (C5), 40.8 (C7), 37.9 (C16), 28.3 (C31). HRMS (ESI) m/z: $[M+H]^+$ calculated for $C_{37}H_{49}N_4O_{10}$: 709.3449, found 709.3436.

General method B for the synthesis of 16-19

To a solution of the benzyl ester protected HDACi-linker conjugate (1 equiv.) in THF, Pd/C (10% wt) was added. The reaction flask was filled with nitrogen and evacuated 3 times using a Shlenk line, before a balloon of hydrogen was added and the resultant mixture stirred vigorously for 4-18 hours. The balloon of hydrogen was removed and the flask was flushed with nitrogen. The reaction mixture was filtered through a glass microfiber filter paper, and the filtrate concentrated *in vacuo* to afford the desired compound.

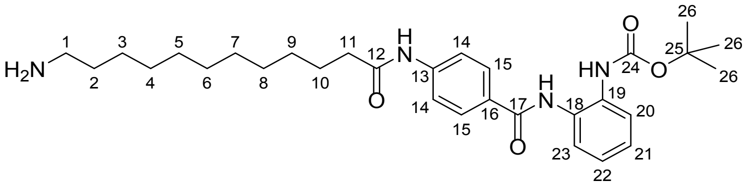

Tert-butyl (2-(4-(12-aminododecanamido)benzamido)phenyl)carbamate (16): Following general method B, N-carboxybenzyl (Cbz) hydrogenation of **12** (73.8 mg, 0.112 mmol) was performed to afford **16** (59.2 mg, 0.112 mmol, 100% yield) as a light brown solid. ^1H NMR (400 MHz, CD_3OD) δ_H ppm 7.94 (d, *J*=8.7 Hz, 2 H, 15-CH), 7.74 (d, *J*=8.7 Hz, 2 H, 14-CH), 7.56 - 7.64 (m, 1 H, 23-CH), 7.40 - 7.46 (m, 1 H, 20-CH), 7.18 - 7.25 (m, 2 H, (21,22)-CH), 2.66 (t, *J*=7.3 Hz, 1 H, 1-CH_2), 2.40 (t, *J*=7.5 Hz, 2 H, 11-CH_2), 1.70 (quin, *J*=7.3 Hz, 2 H, 10-CH_2), 1.51 - 1.63 (m, 2 H, 2-CH_2), 1.50 (s, 9 H, 26-CH_3), 1.29 - 1.39 (m, 14 H, (3-9)-CH_2). ^{13}C NMR (101 MHz, CD_3OD) δ_C ppm 175.1 (C12), 167.9 (C17), 156.4 (C24), 143.9 (C13), 133.2 (C19), 131.8 (C18), 130.3 (C16), 129.7 (C15), 127.5 (C21), 127.3 (C23), 126.4 (C22), 125.7 (C20), 120.4 (C14), 81.9 (C25), 42.4 (C1), 38.2 (C11), 31.5 (C2), 30.8 (alkyl CH_2), 30.75 (alkyl CH_2), 30.7 (alkyl CH_2), 30.65 (alkyl CH_2), 30.6 (alkyl CH_2), 30.5 (alkyl CH_2), 28.8 (C26), 28.1 (C3), 26.9 (C10). HRMS (ESI) m/z: $[M+H]^+$ calculated for $C_{30}H_{45}N_4O_4$: 525.3441, found 525.3448.

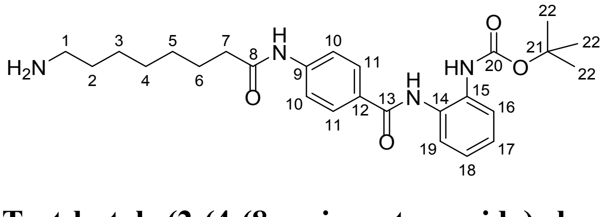

Tert-butyl (2-(4-(8-aminooctanamido) benzamido)phenyl)carbamate (17): Following general method B, Cbz hydrogenation of **13** (303 mg, 0.503 mmol) was performed to afford

17 (241 mg, 0.499 mmol, 99% yield) as a white solid. ^{1}H NMR (400 MHz, CD_3OD) δ_H ppm 7.94 (d, *J*=8.9 Hz, 2 H, 11-CH), 7.74 (d, *J*=8.9 Hz, 2 H, 10-CH), 7.56 - 7.63 (m, 1 H, 19-CH), 7.40 - 7.46 (m, 1 H, 16-CH), 7.18 - 7.26 (m, 2 H, (17,18)-CH), 2.66 (t, *J*=7.1 Hz, 2 H, 1-CH_2), 2.41 (t, *J*=7.5 Hz, 2 H, 7-CH_2), 1.72 (quin, *J*=7.2 Hz, 2 H, 6-CH_2), 1.46 - 1.55 (m, 11 H, 2-CH_2,22-CH_3), 1.33 - 1.43 (m, 6 H, (3-5)-CH_2). ^{13}C NMR (101 MHz, CD_3OD) δ_C ppm 175.1 (C8), 167.9 (C13), 156.5 (C), 143.9 (C9), 133.2 (C15), 131.9 (C14), 130.3 (C12), 129.7 (C11), 127.5 (C16), 127.3 (C17), 126.4 (C19), 125.7 (C18), 120.4 (C10), 81.9 (C21), 42.4 (C1), 38.2 (C7), 33.2 (C2), 30.4 (C3/4/5), 30.35 (C3/4/5), 28.8 (C22), 27.9 (C3/4/5), 26.8 (C6). HRMS (ESI) m/z: $[M+H]^+$ calculated for $C_{26}H_{36}N_4O_4$: 469.2815, found 469.2798.

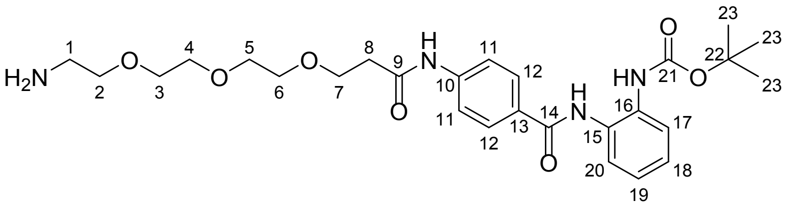

Tert-butyl (2-(4-(3-(2-(2-(2-aminoethoxy)ethoxy)ethoxy)propanamido)benzamido)-phenyl)carbamate (18): Following general method B, Cbz hydrogenation of **14** (284 mg, 0.427 mmol) was performed to afford **18** (224 mg, 0.422 mmol, 99% yield) as a colourless tar. ^{1}H NMR (400 MHz, CD_3OD) δ_H ppm 7.94 (d, *J*=8.9 Hz, 2 H, 12-CH), 7.75 (d, *J*=8.9 Hz, 2 H, 11-CH), 7.56 - 7.64 (m, 1 H, 20-CH), 7.40 - 7.47 (m, 1 H, 17-CH), 7.18 - 7.26 (m, 2 H, (18,19)-CH), 3.83 (t, *J*=6.0 Hz, 2 H, 7-CH_2), 3.64 - 3.66 (m, 4 H, (5,6)-CH_2), 3.61 - 3.64 (m, 2 H, 3/4-CH_2), 3.55 - 3.58 (m, 2 H, 3/4-CH_2), 3.48 t, *J*=5.3 Hz, 2 H, 2-CH_2), 2.77 (t, *J*=5.3 Hz, 2 H, 1-CH_2), 2.67 (t, *J*=6.0 Hz, 2 H, 8-CH_2), 1.50 (s, 9 H, 23-CH_3). ^{13}C NMR (101 MHz, CD_3OD) δ_C ppm 172.8 (C9), 167.9 (C14), 156.4 (C21), 143.8 (C10), 133.2 (C16), 131.8 (C15), 130.4 (C13), 129.7 (C12), 127.5 (C19), 127.3 (C20), 126.4 (C18), 125.7 (C17), 120.5 (C11), 81.9 (C22), 73.0 (C2), 71.7 (alkoxy CH_2), 71.6 (alkoxy CH_2), 71.6 (alkoxy CH_2), 71.4 (alkoxy CH_2), 68.2 (C7), 42.1 (C1), 38.8 (C8), 28.8 (C23). HRMS (ESI) m/z: $[M+H]^+$ calculated for $C_{27}H_{39}N_4O_7$: 531.2819, found 531.2818.

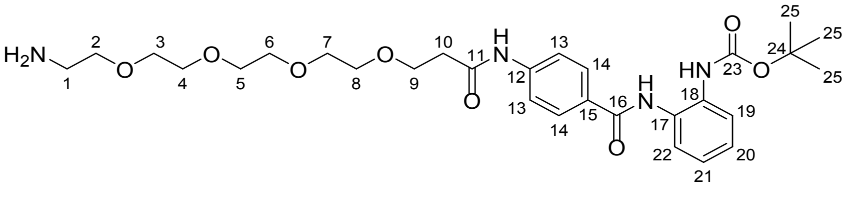

Tert-butyl (2-(4-(1-amino-3,6,9,12-tetraoxapentadecan-15-amido)benzamido)-phenyl)carbamate (19): Following general method B, Cbz hydrogenation of **15** (338 mg,

0.477 mmol) was performed to afford **19** (263 mg, 0.453 mmol, 95% yield) as a pale yellow tar. ^{1}H NMR (400 MHz, CD_3OD) δ_H ppm 7.95 (d, *J*=8.8 Hz, 2 H, 14-CH), 7.77 (d, *J*=8.8 Hz, 2 H, 13-CH), 7.56 - 7.65 (m, 1 H, 22-CH), 7.40 - 7.47 (m, 1 H, 19-CH), 7.18 - 7.26 (m, 2 H, (20,21)-CH), 3.84 (t, *J*=5.9 Hz, 2 H, 9-CH_2), 3.69 - 3.72 (m, 2 H, 2-CH_2), 3.62 - 3.66 (m, 8 H, (5,6,7,8)-CH_2), 3.57 - 3.61 (m, 4 H, (3,4)-CH_2), 3.09 - 3.14 (m, 2 H, 1-CH_2), 2.70 (t, *J*=5.9 Hz, 2 H, 10-CH_2), 1.50 (s, 9 H, 25-CH_3). ^{13}C NMR (101 MHz, CD_3OD) δ_C ppm 172.9 (C11), 167.8 (C16), 156.4 (C23), 143.8 (C12), 133.2 (C18), 131.8 (C17), 130.5 (C15), 129.7 (C14), 127.5 (C21), 127.3 (C22), 126.4 (C20), 125.7 (C19), 120.5 (C13), 81.9 (C24), 71.6 (alkoxy CH_2), 71.5 (alkoxy CH_2), 71.45 (alkoxy CH_2), 71.4 (alkoxy CH_2), 71.35 (alkoxy CH_2), 71.0 (C3), 68.2 (C9), 67.9 (C7), 40.7 (C1), 38.6 (C10), 28.8 (C25). HRMS (ESI) m/z: $[M+H]^+$ calculated for $C_{29}H_{43}N_4O_8$: 575.3081, found 575.3078.

General method C for the synthesis of 20-23

To a solution of idasanutlin (1 equiv.) in dry DMF (1 mL) at 0 °C, DIPEA (3 equiv.) and HATU (1.2 equiv.) were added. The reaction mixture was stirred for 15 minutes, after which a solution of HDACi-linker amine (1 equiv.) in DMF (1 mL) was added slowly and the resultant solution stirred at room temperature for 16 hours. The reaction mixture was diluted in EtOAc (10 mL), then washed with sat. $NaHCO_3$ (2 x 5 mL) and sat. NaCl (2 x 5 mL). The organic layer was dried over $MgSO_4$, filtered, and concentrated *in vacuo* to give the corresponding crude, which was chromatographically purified to afford the desired compound.

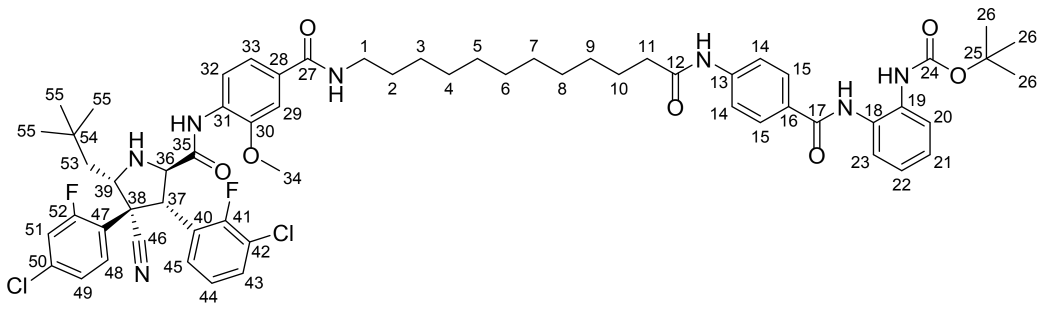

Tert-butyl (2-(4-(12-(4-((2R,3S,4R,5S)-3-(3-chloro-2-fluorophenyl)-4-(4-chloro-2-fluorophenyl)-4-cyano-5-neopentylpyrrolidine-2-carboxamido)-3-methoxybenzamido)dodecanamido)benzamido)phenyl)carbamate (20): Following general method C, **20** was obtained from **idasanutlin** (30.0 mg, 0.049 mmol) and **16** (25.5 mg, 0.049 mmol). The crude product was purified by column chromatography (1-8% MeOH in DCM) to afford **20** (50.0 mg, 0.042 mmol, 86% yield) as a pale yellow solid. ^{1}H NMR (400 MHz, CD_3OD) δ_H ppm 8.35 (d, *J*=8.4 Hz, 1 H, 32-CH), 7.93 (d, *J*=8.8 Hz, 2 H, 15-CH), 7.73 (d, *J*=8.8 Hz, 2 H,

14-CH), 7.68 - 7.71 (m, 1 H, 43-CH), 7.57 - 7.63 (m, 1 H, 23-CH), 7.52 (d, *J*=1.8 Hz, 1 H, 29-CH), 7.40 - 7.45 (m, 2 H, (20,33)-CH), 7.36 - 7.40 (m, 1 H, 44-CH), 7.30 - 7.36 (m, 2 H, (48,51)-CH), 7.17 - 7.25 (m, 4 H, (22,23,45,49)-CH,), 4.75 (d, *J*=8.4 Hz, 1 H, 37-CH), 4.61 (d, *J*=8.4 Hz, 1 H, 36-CH), 4.07 (d, *J*=9.4 Hz, 1 H, 39-CH), 3.96 (s, 3 H, 34-CH_3), 3.36 (t, *J*=7.2 Hz, 2 H, 1-CH_2), 2.38 (t, *J*=7.4 Hz, 2 H, 11-CH_2), 1.65 - 1.73 (m, 3 H, 10-CH_2,53-CH), 1.57 - 1.64 (m, 2 H, 2-CH_2), 1.49 (s, 9 H, 26-CH_3), 1.29 - 1.39 (m, 15 H, 53-CH,(3-9)-CH_2), 1.03 (s, 9 H, 55-CH_3). ^{13}C NMR (101 MHz, CD_3OD) δ_C ppm 175.1 (C12), 173.4 (C35), 169.5 (C27), 167.9 (C17), 161.6 (d, J_{CF}=251.4 Hz, C52), 157.9 (d, J_{CF}=247.6 Hz, C41), 156.4 (C24), 149.9 (C30), 143.9 (C13), 137.5 (d, J_{CF}=11.3 Hz, C50), 133.2 (C19), 132.7 (d, J_{CF}=4.6 Hz, C48), 131.8 (C18), 131.6 (C28), 131.4 (C44), 131.2 (C31), 130.3 (C16), 129.7 (C15), 129.5 (d, J_{CF}=1.9 Hz, C43), 127.5 (C21/22), 127.3 (C23), 127.1 (d, J_{CF}=13.7 Hz, C40), 126.6 (d, J_{CF}=3.1 Hz, C49), 126.4 (C21/22), 126.1 (d, J_{CF}=4.6 Hz, C45), 125.7 (C20), 122.1 (d, J_{CF}=19.3 Hz, C42),121.2 (C33), 120.4 (C14), 120.3 (d, J_{CF}=10.0 Hz C47), 119.4 (C32), 118.9 (d, J_{CF}=27.5 Hz, C51), 118.6 (C46), 110.6 (C29), 81.9 (C25), 66.4 (d, J_{CF}=7.4 Hz, C38), 66.0 (C36), 64.6 (d, J_{CF}=7.8 Hz, C39), 56.6 (C34), 51.6 (d, J_{CF}=3.2 Hz, C37), 46.0 (C53), 41.3 (C1), 38.2 (C11), 31.4 (C54), 30.75 (alkyl CH_2), 30.7 (alkyl CH_2), 30.65 (alkyl CH_2), 30.6 (C2), 30.55 (2 x alkyl CH_2), 30.5 (C55), 30.4 (alkyl CH_2), 28.8 (C26), 28.2 (C3), 26.9 (C10). ^{19}F NMR (376 MHz, CD_3OD) δ_F ppm -109.4 (52-CF), -121.4 (41-CF). HRMS (ESI) m/z: $[M+H]^+$ calculated for $C_{61}H_{72}{}^{35}Cl_2F_2N_7O_7$: 1122.4838, found 1122.4852; $C_{61}H_{72}{}^{35}Cl^{37}ClF_2N_7O_7$: 1124.4809, found 1124.4849; $C_{61}H_{72}{}^{37}Cl_2F_2N_7O_7$: 1126.4839, found 1126.4861 (Mass accuracy at 7.3pppm).

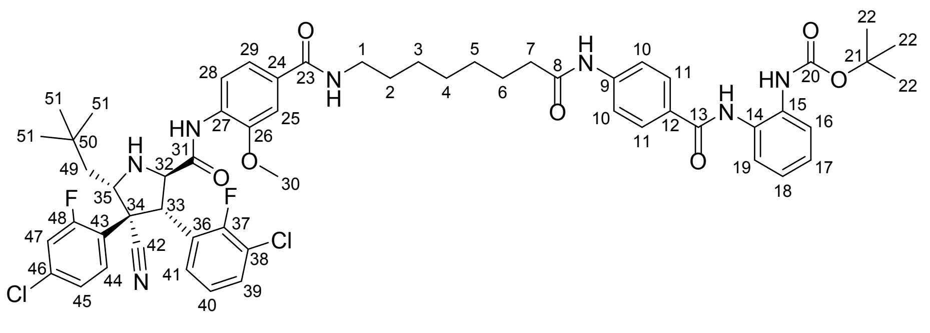

Tert-butyl (2-(4-(8-(4-((2R,3S,4R,5S)-3-(3-chloro-2-fluorophenyl)-4-(4-chloro-2-fluorophenyl)-4-cyano-5-neopentylpyrrolidine-2-carboxamido)-3-methoxybenzamido)octanamido)benzamido)phenyl)carbamate (21): Following general method C, **21** was obtained from **idasanutlin** (30.0 mg, 0.049 mmol) and **17** (22.8 mg, 0.049 mmol). The crude product was purified by column chromatography (1-8% MeOH in DCM) to afford **21** (46.2 mg, 0.041 mmol, 84% yield) as a pale yellow solid. ^{1}H NMR (400 MHz, CD_3OD) δ_H ppm 8.34 (d, *J*=8.4 Hz, 1 H, 28-CH), 7.92 (d, *J*=8.8 Hz, 2 H, 11-CH), 7.71 (d, *J*=8.8 Hz, 2 H, 10-CH),

7.67 - 7.70 (m, 1 H, 39-CH), 7.57 - 7.64 (m, 1 H, 19-CH), 7.51 (d, *J*=1.8 Hz, 1 H, 25-CH), 7.40 - 7.45 (m, 2 H, (16,29)-CH), 7.35 - 7.40 (m, 1 H, 40-CH), 7.30 - 7.35 (m, 2 H, (44,47)-CH), 7.17 - 7.25 (m, 4 H, (18,19,41,45)-CH), 4.75 (d, *J*=8.4 Hz, 1 H, 33-CH), 4.60 (d, *J*=8.4 Hz, 1 H, 32-CH), 4.06 (d, *J*=9.4 Hz, 1 H, 35-CH), 3.95 (s, 3 H, 30-CH), 3.36 (t, *J*=7.2 Hz, 2 H, 1-CH_2), 2.39 (t, *J*=7.5 Hz, 2 H, 7-CH_2), 1.66 - 1.76 (m, 3 H, 6-CH_2,49-CH), 1.58 - 1.65 (m, 2 H, 2-CH_2), 1.48 (s, 9 H, 22-CH_3), 1.32 - 1.44 (m, 7 H, (3-5)-CH_2,49-CH), 1.02 (s, 9 H, 51-CH_3). ^{13}C NMR (101 MHz, CD_3OD) δ_C ppm 175.0 (C8), 173.4 (C31), 169.5 (C23), 167.8 (C13), 161.5 (d, J_{CF}=251.2 Hz, C48), 157.9 (d, J_{CF}=247.8 Hz, C37), 156.4 (C20), 149.9 (C26), 143.9 (C9), 137.5 (d, J_{CF}=11.3 Hz, C46), 133.1 (C15), 132.7 (d, J_{CF}=4.4 Hz, C44), 131.8 (C14), 131.6 (C24), 131.4 (C40), 131.1 (C27), 130.3 (C12), 129.7 (C11), 129.5 (d, J_{CF}=2.3 Hz, C39), 127.5 (C17/18), 127.2 (C19), 127.1 (d, J_{CF}=13.5 Hz, C36), 126.6 (d, J_{CF}=3.1 Hz, C45), 126.4 (C43), 126.1 (d, J_{CF}=4.6 Hz, C41), 125.7 (C16), 122.1 (d, J_{CF}=19.1 Hz, C38), 121.2 (C29), 120.4 (C10), 120.3 (d, J_{CF}=10.0 Hz, C43), 119.4 (C28), 118.9 (d, *J*=27.5 Hz, C47), 118.6 (C42), 110.6 (C25), 81.9 (C21), 66.4 (d, J_{CF}=7.2 Hz, C34), 66.0 (C32), 64.6 (d, J_{CF}=7.8 Hz, C35), 56.5 (C30), 51.5 (d, J_{CF}=3.2 Hz, C33), 46.0 (C49), 41.2 (C1), 38.2 (C7), 31.4 (C50), 30.6 (C2), 30.5 (C51), 30.3 (C4/5), 30.2 (C4/5), 28.8 (C22), 28.0 (C3), 26.7 (C6). ^{19}F NMR (376 MHz, CD_3OD) δ_F ppm -109.4 (48-CF), -121.4 (37-CF). HRMS (ESI) m/z: $[M+H]^+$ calculated for $C_{57}H_{63}{}^{35}Cl_2F_2N_7O_7$: 1066.4212, found 1066.4214; $C_{57}H_{63}{}^{35}Cl^{37}ClF_2N_7O_7$: 1068.4183, found 1068.4196; $C_{57}H_{63}{}^{37}Cl_2F_2N_7O_7$: 1070.4153, found 1070.4143.

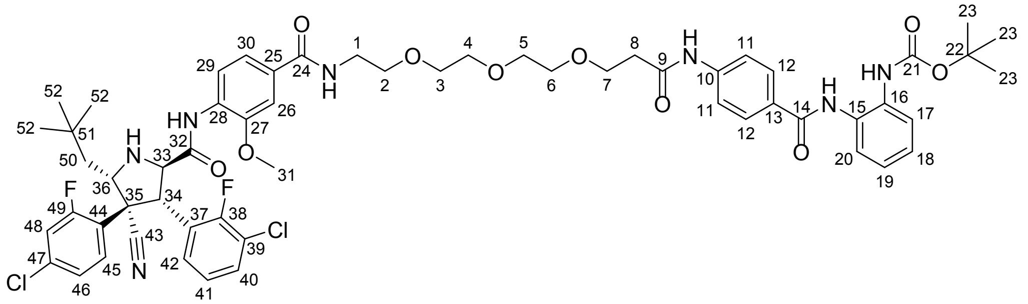

Tert-butyl (2-(4-(1-(4-((2R,3S,4R,5S)-3-(3-chloro-2-fluorophenyl)-4-(4-chloro-2-fluorophenyl)-4-cyano-5-neopentylpyrrolidine-2-carboxamido)-3-methoxyphenyl)-1-oxo-5,8,11-trioxa-2-azatetradecan-14-amido)benzamido)phenyl)carbamate (22): Following general method C, **22** was obtained from idasanutlin (30.0 mg, 0.049 mmol) and **18** (25.8 mg, 0.049 mmol). The crude product was purified by column chromatography (1-10% MeOH in DCM) to afford **22** (43.9 mg, 0.037 mmol, 75% yield) as a pale yellow solid. ^{1}H NMR (400 MHz, CD_3OD) δ_H ppm 8.34 (d, *J*=8.4 Hz, 1 H, 29-CH), 7.90 (d, *J*=8.8 Hz, 2 H, 12-CH), 7.65 - 7.72 (m, 3 H, (11,40)-CH), 7.60 (dd, *J*=7.6, 1.8 Hz, 1 H, 20-CH), 7.50 (d, *J*=1.8

Hz, 1 H, 26-CH), 7.41 - 7.46 (m, 2 H, (17,30)-CH), 7.35 - 7.40 (m, 1 H, 41-CH), 7.30 - 7.35 (m, 2 H, (45,48)-CH), 7.17 - 7.24 (m, 4 H, (19,20,42,46)-CH), 4.75 (d, *J*=8.4 Hz, 1 H, 34-CH), 4.60 (d, *J*=8.4 Hz, 1 H, 33-CH), 4.06 (d, *J*=9.3 Hz, 1 H, 36-CH), 3.93 (s, 3 H, 31-CH_3), 3.78 (t, *J*=6.0 Hz, 2 H, 7-CH_2), 3.59 - 3.65 (m, 10 H, (2-6)-CH_2), 3.53 - 3.57 (m, 2 H, 1-CH_2), 2.61 (t, *J*=6.0 Hz, 2 H, 8-CH_2), 1.69 (dd, *J*=14.3, 9.3 Hz, 1 H, 50-CH), 1.48 (s, 9 H, 23-CH_3), 1.32 - 1.37 (m, 1 H, 50-CH), 1.03 (s, 9 H, 52-CH_3). ^{13}C NMR (101 MHz, CD_3OD) δ_C ppm 173.4 (C32), 172.7 (C9), 169.6 (C24), 167.8 (C14), 161.5 (d, J_{CF}=250.8 Hz, C49), 157.9 (d, J_{CF}=247.6 Hz, C38), 156.4 (C21), 149.8 (C27), 143.8 (C10), 137.5 (d, J_{CF}=11.3 Hz, C47), 133.1 (C16), 132.7 (d, J_{CF}=4.8 Hz, C45), 131.8 (C15), 131.4 (C41), 131.3 (C25), 131.2 (C28), 130.4 (C13), 129.7 (C12), 129.5 (d, J_{CF}=2.1 Hz, C40), 127.5 (C18/19), 127.3 (C20), 127.1 (d, J_{CF}=13.5 Hz, C37), 126.6 (d, J_{CF}=2.9 Hz, C46), 126.3 (C18/19), 126.1 (d, J_{CF}=4.6 Hz, C42), 125.6 (C17), 122.0 (d, J_{CF}=19.1 Hz, C39), 121.3 (C30), 120.4 (C11), 120.3 (d, J_{CF}=10.0 Hz, C44), 119.4 (C29), 118.9 (d, J_{CF}=27.5 Hz, C48), 118.6 (C43), 110.7 (C26), 81.9 (C22), 71.7 (alkoxy CH_2), 71.6 (2 x alkoxy CH_2), 71.5 (alkoxy CH_2), 70.7 (C2), 68.2 (C7), 66.4 (d, J_{CF}=7.4 Hz, C35), 66.0 (C33), 64.6 (d, J_{CF}=7.8 Hz, C36), 56.6 (C31), 51.6 (d, *J*=3.8 Hz, C34), 46.0 (C50), 41.2 (C1), 38.8 (C8), 31.4 (C51), 30.5 (C52), 28.8 (C23). ^{19}F NMR (376 MHz, CD_3OD) δ_F ppm -109.4 (49-CF), -121.4 (38-CF). HRMS (ESI) m/z calculated for $C_{58}H_{66}{}^{35}Cl_2F_2N_7O_{10}$: 1128.4216, found 1128.4215; $C_{58}H_{66}{}^{35}Cl^{37}ClF_2N_7O_{10}$: 1130.4187, found 1130.4197; $C_{58}H_{66}{}^{37}Cl_2F_2N_7O_{10}$: 1132.4157, found 1132.4182.

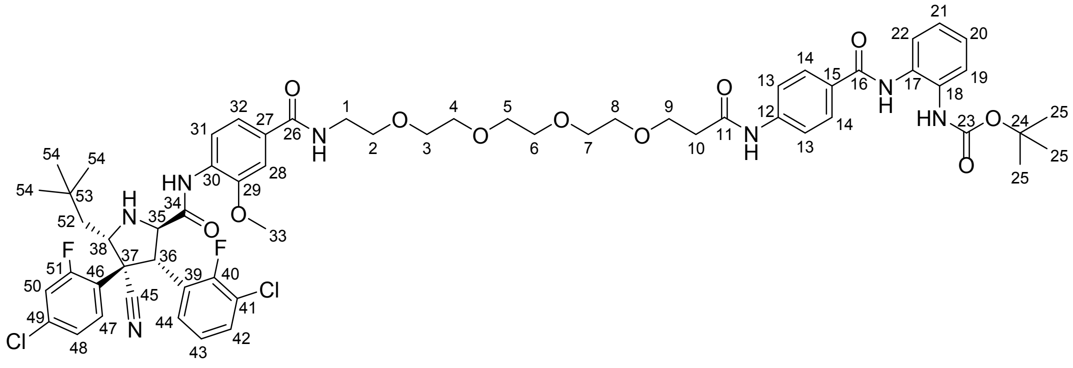

Tert-butyl (2-(4-(1-(4-((2R,3S,4R,5S)-3-(3-chloro-2-fluorophenyl)-4-(4-chloro-2-fluorophenyl)-4-cyano-5-neopentylpyrrolidine-2-carboxamido)-3-methoxyphenyl)-1-oxo-5,8,11,14-tetraoxa-2-azaheptadecan-17-amido)benzamido)phenyl)carbamate (23): Following general method C, **23** was obtained from idasanutlin (30.0 mg, 0.049 mmol) and **19** (28.0 mg, 0.049 mmol). The crude product was purified by column chromatography (1-10% MeOH in DCM) to afford **23** (42.3 mg, 0.034 mmol, 70% yield) as a pale yellow solid. ^{1}H NMR (400 MHz, CD_3OD) δ_H ppm 8.35 (d, *J*=8.4 Hz, 1 H, 31-CH), 7.92 (d, *J*=8.8 Hz, 2 H, 14-

CH), 7.66 - 7.74 (m, 3 H, (13,42)-CH), 7.60 (dd, *J*=7.5, 1.9 Hz, 1 H, 22-CH), 7.52 (d, *J*=1.8 Hz, 1 H, 28-CH), 7.41 - 7.45 (m, 2 H, (19,32)-CH), 7.35 - 7.40 (m, 1 H, 43-CH), 7.31 - 7.35 (m, 2 H, (47,50)-CH), 7.18 - 7.24 (m, 4 H, (21,22,44,48)-CH), 4.75 (d, *J*=8.4 Hz, 1 H, 36-CH), 4.60 (d, *J*=8.4 Hz, 1 H, 35-CH), 4.06 (d, *J*=9.4 Hz, 1 H, 38-CH), 3.94 (s, 3 H, 33-CH_3), 3.78 (t, *J*=6.0 Hz, 2 H, 9-CH_2), 3.55 - 3.64 (m, 16 H, (1-8)-CH_2), 2.62 (t, *J*=6.0 Hz, 2 H, 10-CH_2), 1.69 (dd, *J*=14.3, 9.4 Hz, 1 H, 52-CH), 1.48 (s, 9 H, 25-CH_3), 1.32 - 1.38 (m, 1 H, 52-CH), 1.03 (s, 9 H, 54-CH_3). ^{13}C NMR (101 MHz, CD_3OD) δ_C ppm 173.4 (C34), 172.7 (C11), 169.6 (C26), 167.8 (C16), 161.5 (d, J_{CF}=251.0 Hz, C51), 157.9 (d, J_{CF}=247.6 Hz, C40), 156.4 (C23), 149.8 (C29), 143.8 (C12), 137.5 (d, J_{CF}=11.4 Hz, C49), 133.1 (C18), 132.7 (d, J_{CF}=4.6 Hz, C47), 131.8 (C17), 131.4 (C43), 131.3 (C27), 131.2 (C30), 130.4 (C15), 129.7 (C14), 129.5 (d, J_{CF}=1.9 Hz, C42), 127.5 (C20/21), 127.3 (C22), 127.1 (d, J_{CF}=13.4 Hz, C39), 126.6 (d, J_{CF}=3.1 Hz, C48), 126.4 (C20/21), 126.1 (d, J_{CF}=4.6 Hz, C44), 125.6 (C19), 122.0 (d, *J*=18.9 Hz, C41), 121.4 (C32), 120.5 (C13), 120.3 (d, J_{CF}=10.0 Hz, C46), 119.4 (C31), 118.9 (d, J_{CF}=27.5 Hz, C50), 118.6 (C45), 110.7 (C28), 81.9 (C26), 71.7 (2 x alkoxy CH_2), 71.65 (alkoxy CH_2), 71.6 (alkoxy CH_2), 71.55 (alkoxy CH_2), 71.4 (alkoxy CH_2), 70.7 (C2), 68.2 (C9), 66.4 (d, J_{CF}=7.6 Hz, C37), 66.0 (C35), 64.6 (d, J_{CF}=8.2 Hz, C38), 56.6 (C33), 51.6 (d, J_{CF}=4.0 Hz, C36), 46.0 (C52), 41.2 (C1), 38.8 (C10), 31.4 (C53), 30.5 (C54), 28.8 (C25). ^{19}F NMR (376 MHz, CD_3OD) δ_F ppm -109.4 (51-CF), -121.4 (40-CF). HRMS (ESI) m/z calculated for $C_{60}H_{70}{}^{35}Cl_2F_2N_7O_{11}$: 1172.4478, found 1172.4458; $C_{60}H_{70}{}^{35}Cl^{37}ClF_2N_7O_{11}$: 1174.4449, found 1174.4442; $C_{60}H_{70}{}^{37}Cl_2F_2N_7O_{11}$: 1176.4419, found 1176.4460.

General method D for the synthesis of 1-4

TFA (0.4 mL or 20 equiv.) was added to a stirring solution of Boc-protected PROTAC (1 equiv.) in DCM (2 mL) and the resulting reaction mixture stirred at room temperature for 4-6 hours. The reaction mixture was concentrated *in vacuo*, dissolved in MeOH (2 mL), agitated in MP-carbonate resin (3.02 mmol/g loading capacity) for 2-3 hours and then filtered. The filtrate was concentrated *in vacuo* and the resulting solid dissolved in MeCN:H_2O (1:1) and lyophilised to remove residual TFA impurities, affording the final PROTAC.

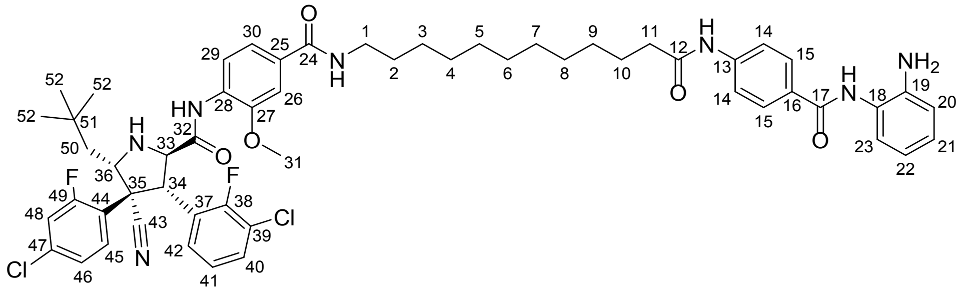

(2R,3S,4R,5S)-N-(4-((12-((4-((2-aminophenyl)carbamoyl)phenyl)amino)-12-oxododecyl)carbamoyl)-2-methoxyphenyl)-3-(3-chloro-2-fluorophenyl)-4-(4-chloro-2-fluorophenyl)-4-cyano-5-neopentylpyrrolidine-2-carboxamide (1): Following general method D, Boc deprotection of **20** (50.0 mg, 0.042 mmol) was performed to afford **1** (39.7 mg, 0.038 mmol, 91% yield) as a pale yellow solid. Prior to biological evaluation the PROTAC was further purified by column chromatography (alumina, 1-10% MeOH in DCM). ^{1}H NMR (400 MHz, CD_3OD) δ_H ppm 8.35 (d, *J*=8.4 Hz, 1 H, 29-CH), 7.95 (d, *J*=8.6 Hz, 2 H, 15-CH), 7.72 (d, *J*=8.8 Hz, 3 H, (14,40)-CH), 7.52 (d, *J*=1.8 Hz, 1 H, 26-CH), 7.42 (dd, *J*=8.4, 1.8 Hz, 1 H, 30-CH), 7.36 - 7.41 (m, 1 H, 41-CH), 7.31 - 7.36 (m, 2 H, (45,48)-CH), 7.20 - 7.25 (m, 2 H, (42,46)-CH), 7.18 (dd, *J*=7.8, 1.3 Hz, 1 H, 23-CH), 7.07 (app. td, *J*=7.8, 1.3 Hz, 1 H, 21-CH), 6.90 (dd, *J*=7.8, 1.3 Hz, 1 H, 20-CH), 6.76 (app. td, *J*=7.8, 1.3 Hz, 1 H, 22-CH), 4.76 (d, *J*=8.4 Hz, 1 H, 34-CH), 4.62 (d, *J*=8.4 Hz, 1 H, 33-CH), 4.07 (d, *J*=9.4 Hz, 1 H, 36-CH), 3.96 (s, 3 H, 31-CH_3), 3.36 (t, *J*=7.2 Hz, 2 H, 1-CH_2), 2.39 (t, *J*=7.4 Hz, 2 H, 11-CH_2), 1.65 - 1.73 (m, 3 H, 10-CH_2,50-CH), 1.57 - 1.64 (m, 2 H, 2-CH_2), 1.30 - 1.39 (m, 15 H, 50-CH,(3-9)-CH_2), 1.03 (s, 9 H, 52-CH_3). ^{13}C NMR (101 MHz, CD_3OD) δ_C ppm 175.1 (C12), 173.5 (C32), 169.5 (C24), 168.4 (C17), 161.6 (d, J_{CF}=251.2 Hz, C49), 157.9 (d, J_{CF}=247.8 Hz, C38), 149.9 (C27), 143.9 (C19), 143.7 (C13), 137.5 (d, J_{CF}=11.3 Hz, C47), 132.7 (d, J_{CF}=4.6 Hz, C45), 131.6 (C25), 131.4 (C41), 131.1 (C28), 130.5 (C16), 129.9 (C15), 129.5 (d, J_{CF}=2.3 Hz, C40), 128.6 (C21), 127.8 (C23), 127.1 (d, J_{CF}=13.5 Hz, C37), 126.6 (d, J_{CF}=3.2 Hz, C46), 126.1 (d, J_{CF}=4.6 Hz, C42), 125.6 (C18), 122.1 (d, J_{CF}=18.9 Hz, C39), 121.2 (C30), 120.4 (C14), 120.3 (d, J_{CF}=10.0 Hz, C44), 119.8 (C22), 119.4 (C29), 118.95 (d, J_{CF}=27.5 Hz, C48), 118.9 (C20), 118.6 (C43), 110.6 (C26), 66.4 (d, J_{CF}=7.4 Hz, C35), 66.0 (C33), 64.6 (d, J_{CF}=7.8 Hz, C36), 56.6 (C31), 51.6 (d, J_{CF}=3.1 Hz, C34), 46.0 (C50), 41.3 (C1), 38.2 (C11), 31.4 (C51), 30.75 (alkyl CH_2), 30.7 (alkyl CH_2), 30.65 (alkyl CH_2), 30.6 (alkyl CH_2), 30.55 (2 x alkyl CH_2), 30.5 (C52), 30.4 (alkyl CH_2), 28.2 (C3), 26.9 (C10). ^{19}F NMR (376 MHz, CD_3OD) δ_C ppm -109.5 (49-CF), -121.5 (38-CF). HRMS (ESI) m/z: $[M+H]^+$ calculated for $C_{56}H_{64}{}^{35}Cl_2F_2N_7O_5$: 1022.4314, found 1022.4326; $C_{56}H_{64}{}^{35}Cl^{37}ClF_2N_7O_5$: 1024.4285, found 1024.4319; $C_{56}H_{64}{}^{37}Cl_2F_2N_7O_5$: 1026.4255, found 1026.4270.

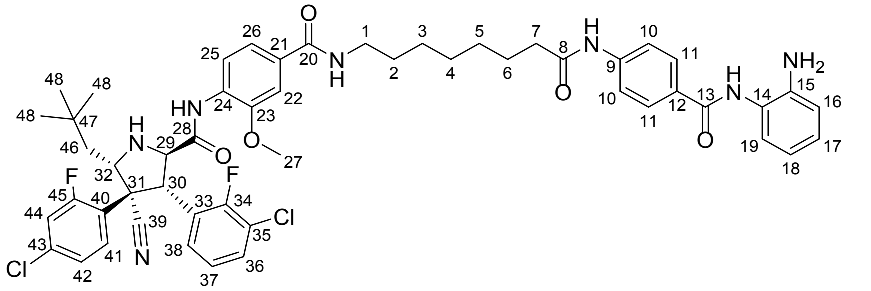

(2R,3S,4R,5S)-N-(4-((12-((4-((2-aminophenyl)carbamoyl)phenyl)amino)-12-oxododecyl)carbamoyl)-2-methoxyphenyl)-3-(3-chloro-2-fluorophenyl)-4-(4-chloro-2-fluorophenyl)-4-cyano-5-neopentylpyrrolidine-2-carboxamide (2): Following general method D, Boc deprotection of **21** (46.2 mg, 0.041 mmol) was performed to afford **2** (32.8 mg, 0.034 mmol, 82% yield) as a pale yellow solid. Prior to biological evaluation the PROTAC was further purified by column chromatography (alumina, 1-10% MeOH in DCM). ^{1}H NMR (400 MHz, CD_3OD) δ_H ppm 8.34 (d, *J*=8.4 Hz, 1 H, 25-CH), 7.94 (d, *J*=8.7 Hz, 2 H, 11-CH), 7.66 - 7.74 (m, 3 H, (10,36)-CH), 7.52 (d, *J*=1.9 Hz, 1 H, 22-CH), 7.42 (dd, *J*=8.4, 1.9 Hz, 1 H, 26-CH), 7.36 - 7.40 (m, 1 H, 37-CH), 7.31 - 7.36 (m, 2 H, (41,44)-CH), 7.20 - 7.26 (m, 2 H, (38,42)-CH), 7.18 (d, *J*=7.8, 1.3 Hz, 1 H, 19-CH), 7.07 (app. td, *J*=7.8, 1.3 Hz, 1 H, 17-CH), 6.90 (dd, *J*=7.8, 1.3 Hz, 1 H, 16-CH), 6.77 (app. td, *J*=7.8, 1.3 Hz, 1 H, 18-CH), 4.75 (d, *J*=8.4 Hz, 1 H, 30-CH), 4.61 (dd, *J*=8.4 Hz, 1 H, 29-CH), 4.06 (d, *J*=9.2 Hz, 1 H, 32-CH), 3.96 (s, 3 H, 27-CH_3), 3.37 (t, *J*=7.1 Hz, 2 H, 1-CH_2), 2.40 (t, *J*=7.4 Hz, 2 H, 7-CH_2), 1.66 - 1.76 (m, 3 H, 6-CH_2,46-CH), 1.59 - 1.66 (m, 2 H, 2-CH_2), 1.38 - 1.46 (m, 6 H, (3-5)-CH_2), 1.33 - 1.37 (m, 1 H, 46-CH), 1.03 (s, 9 H, 48-CH_3). ^{13}C NMR (101 MHz, CD_3OD) δ_C ppm 175.1 (C8), 173.4 (C28), 169.6 (C20), 168.4 (C13), 161.5 (d, J_{CF}=251.0 Hz, C45), 157.9 (d, J_{CF}=247.6 Hz, C34), 149.9 (C23), 143.9 (C15), 143.6 (C9), 137.5 (d, J_{CF}=11.3 Hz, C43), 132.7 (d, J_{CF}=4.6 Hz, C41), 131.6 (C21), 131.4 (C37), 131.2 (C24), 130.5 (C12), 129.9 (C11), 129.5 (d, J_{CF}=2.1 Hz, C36), 128.6 (C17), 127.8 (C19), 127.1 (d, J_{CF}=13.7 Hz, C33), 126.6 (d, J_{CF}=3.1 Hz, C42), 126.1 (d, J_{CF}=4.6 Hz, C38), 125.6 (C14), 122.1 (d, J_{CF}=19.1 Hz, C35), 121.2 (C26), 120.4 (C10), 120.3 (d, J_{CF}=10.0 Hz, C40), 119.9 (C18), 119.4 (C25), 118.95 (d, J_{CF}=27.5 Hz, C44), 118.9 (C16), 118.6 (C39), 110.6 (C22), 66.4 (d, J_{CF}=7.4 Hz, C31), 66.0 (C29), 64.6 (d, J_{CF}=7.6 Hz, C32), 56.5 (C27), 51.6 (d, J_{CF}=3.8 Hz, C30), 46.0 (C46), 41.2 (C1), 38.2 (C7), 31.4 (C47), 30.6 (C2), 30.5 (C48), 30.3 (C4/5), 30.2 (C4/5), 28.0 (C3), 26.8 (C10). ^{19}F NMR (376 MHz, CD_3OD) δ_F ppm -109.4 (45-CF), -121.4 (34-CF). HRMS (ESI) m/z: $[M+H]^+$ calculated for $C_{52}H_{56}{}^{35}Cl_2F_2N_7O_5$: 966.3688, found 966.3680; $C_{52}H_{56}{}^{35}Cl^{37}ClF_2N_7O_5$: 968.3659, found 968.3671; $C_{52}H_{56}{}^{37}Cl_2F_2N_7O_5$: 970.3629, found 970.3613.

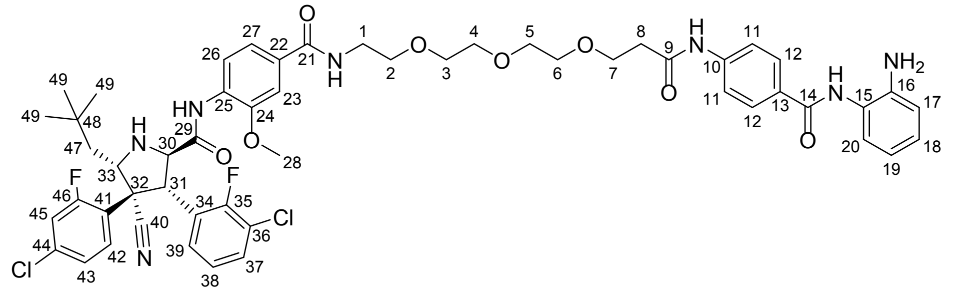

(2R,3S,4R,5S)-N-(4-((8-((4-((2-aminophenyl)carbamoyl)phenyl)amino)-8-oxo-octyl)carbamoyl)-2-methoxyphenyl)-3-(3-chloro-2-fluorophenyl)-4-(4-chloro-2-fluorophenyl)-4-cyano-5-neopentylpyrrolidine-2-carboxamide (3): Following general method D, Boc deprotection of **22** (43.9 mg, 0.037 mmol) was performed to afford **3** (38.7 mg, 0.037 mmol, 100% yield) as a pale yellow solid. Prior to biological evaluation the PROTAC was further purified by column chromatography (alumina, 1-10% MeOH in DCM). ^{1}H NMR (400 MHz, CD_3OD) δ_H ppm 8.34 (d, *J*=8.4 Hz, 1 H, 26-CH), 7.92 (d, *J*=8.7 Hz, 2 H, 12-CH), 7.66 - 7.72 (m, 3 H, (11,37)-CH), 7.51 (d, *J*=1.8 Hz, 1 H, 23-CH), 7.43 (dd, *J*=8.4, 1.8 Hz, 1 H, 27-CH), 7.35 - 7.39 (m, 1 H, 38-CH), 7.30 - 7.35 (m, 2 H, (42,45)-CH), 7.20 - 7.24 (m, 2 H, (39,43)-CH), 7.18 (dd, *J*=7.8, 1.3 Hz, 1 H, 20-CH), 7.06 (app. td, *J*=7.8, 1.3 Hz, 1 H, 18-CH), 6.89 (dd, *J*=7.8, 1.3 Hz, 1 H, 17-CH), 6.75 (app. td, *J*=7.8, 1.3 Hz, 1 H, 19-CH), 4.75 (d, *J*=8.4 Hz, 1 H, 31-CH), 4.60 (d, *J*=8.4 Hz, 1 H, 30-CH), 4.06 (d, *J*=9.4 Hz, 1 H, 33-CH), 3.93 (s, 3 H, 28-CH_3), 3.78 (t, *J*=6.0 Hz, 2 H, 7-CH_2), 3.58 - 3.65 (m, 10 H, (2-6)-CH_2), 3.52 - 3.56 (m, 2 H, 1-CH_2), 2.60 (t, *J*=6.0 Hz, 2 H, 8-CH_2), 1.69 (dd, *J*=14.1, 9.4 Hz, 1 H, 47-CH), 1.33 - 1.37 (m, 1 H, 47-CH), 1.02 (s, 9 H, 49-CH_3). ^{13}C NMR (101 MHz, CD_3OD) δ_C ppm 173.4 (C29), 172.6 (C9), 169.6 (C21), 168.3 (C17), 161.5 (d, J_{CF}=251.2 Hz, C46), 157.9 (d, J_{CF}=247.6 Hz, C35), 149.9 (C24), 143.9 (C16), 143.5 (C10), 137.5 (d, J_{CF}=11.4 Hz, C44), 132.7 (d, J_{CF}=4.4 Hz, C42), 131.4 (C38), 131.3 (C22), 131.2 (C25), 130.6 (C13), 129.9 (C12), 129.5 (d, J_{CF}=2.1 Hz, C37), 128.6 (C18), 127.8 (C20), 127.1 (d, J_{CF}=13.7 Hz, C34), 126.6 (d, J_{CF}=3.2 Hz, C43), 126.1 (d, J_{CF}=4.6 Hz, C39), 125.6 (C15), 122.0 (d, J_{CF}=19.1 Hz, C36), 121.3 (C27), 120.4 (C11), 120.3 (d, J_{CF}=10.0 Hz, C41), 119.8 (C19), 119.4 (C26), 118.95 (d, J_{CF}=27.5 Hz, C45), 118.9 (C17), 118.6 (C40), 110.7 (C23), 71.7 (alkoxy CH_2), 71.65 (alkoxy CH_2), 71.6 (alkoxy CH_2), 71.5 (alkoxy CH_2), 70.7 (C2), 68.2 (C7), 66.3 (d, J_{CF}=7.2 Hz, C32), 66.0 (C30), 64.6 (d, J_{CF}=7.8 Hz, C33), 56.6 (C28), 51.6 (d, J_{CF}=3.2 Hz, C31), 45.9 (C47), 41.2 (C1), 38.8 (C8), 31.4 (C48), 30.5 (C49). ^{19}F NMR (376 MHz, CD_3OD) δ ppm -109.4 (46-CF), -121.4 (35-CF). HRMS (ESI) m/z: $[M+H]^+$ calculated for $C_{53}H_{58}{}^{35}Cl_2F_2N_7O_8$: 1028.3692, found 1028.3677; $C_{53}H_{58}{}^{35}Cl^{37}ClF_2N_7O_8$: 1030.3662, found 1030.3671; $C_{53}H_{58}{}^{37}Cl_2F_2N_7O_8$: 1032.3633, found 1032.3676.

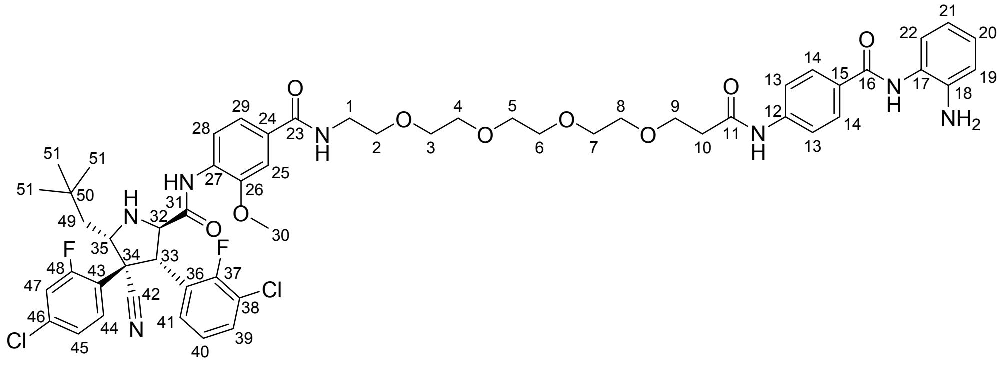

(2R,3S,4R,5S)-N-(4-((15-((4-((2-aminophenyl)carbamoyl)phenyl)amino)-15-oxo-3,6,9,12-tetraoxapentadecyl)carbamoyl)-2-methoxyphenyl)-3-(3-chloro-2-fluorophenyl)-4-(4-chloro-2-fluorophenyl)-4-cyano-5-neopentylpyrrolidine-2-carboxamide (4): Following general method D, Boc deprotection of **23** (42.3 mg, 0.037 mmol) was performed to afford **4** (36.1 mg, 0.033 mmol, 90% yield) as a pale yellow solid. Prior to biological evaluation the PROTAC was further purified by column chromatography (alumina, 1-10% MeOH in DCM). ^{1}H NMR (400 MHz, CD_3OD) δ_H ppm 8.35 (d, *J*=8.4 Hz, 1 H, 28-CH), 7.94 (d, *J*=8.7 Hz, 2 H, 14-CH), 7.67 - 7.74 (m, 3 H, (13,39)-CH), 7.52 (d, *J*=1.8 Hz, 1 H, 25-CH), 7.43 (dd, *J*=8.4, 1.8 Hz, 1 H, 29-CH), 7.36 - 7.40 (m, 1 H, 40-CH), 7.31 - 7.36 (m, 2 H, (44,47)-CH), 7.20 - 7.25 (m, 2 H, (41,45)-CH), 7.18 (dd, *J*=7.8, 1.3 Hz, 1 H, 22-CH), 7.06 (app. td, *J*=7.8, 1.3 Hz, 1 H, 20-CH), 6.89 (dd, *J*=7.8, 1.3 Hz, 1 H, 19-CH), 6.75 (app. td, *J*=7.8, 1.3 Hz, 1 H, 21-CH), 4.75 (d, *J*=8.4 Hz, 1 H, 33-CH), 4.61 (d, *J*=8.34 Hz, 1 H, 32-CH), 4.06 (d, *J*=9.4 Hz, 1 H, 35-CH), 3.95 (s, 3 H, 30-CH_3), 3.78 (t, *J*=6.0 Hz, 2 H, 9-CH_2), 3.61 - 3.64 (m, 2 H, 2-CH_2), 3.55 - 3.60 (m, 14 H, (1,(3-8))-CH_2), 2.61 (t, *J*=6.0 Hz, 2 H, 10-CH_2), 1.69 (dd, *J*=14.3, 9.4 Hz, 1 H, 49-CH), 1.33 - 1.38 (m, 1 H, 49-CH), 1.03 (s, 9 H, 51-CH_3). ^{13}C NMR (101 MHz, CD_3OD) δ_C ppm 173.4 (C31), 172.7 (C11), 169.6 (C23), 168.3 (C19), 161.5 (d, J_{CF}=250.8 Hz, C48), 157.9 (d, J_{CF}=247.6 Hz, C37), 149.9 (C26), 143.9 (C18), 143.5 (C12), 137.5 (d, J_{CF}=11.4 Hz, C46), 132.7 (d, J_{CF}=4.6 Hz, C44), 131.4 (C40), 131.3 (C24), 131.2 (C27), 130.6 (C15), 129.9 (C14), 129.5 (d, J_{CF}=2.3 Hz, C39), 128.6 (C20), 127.8 (C22), 127.1 (d, J_{CF}=13.5 Hz, C36), 126.6 (d, J_{CF}=3.2 Hz, C45), 126.1 (d, J_{CF}=4.6 Hz, C41), 125.6 (C17), 122.1 (d, J_{CF}=19.1 Hz, C38), 121.4 (C29), 120.4 (C13), 120.4 (d, J_{CF}=9.5 Hz, C43), 119.8 (C21), 119.4 (C28), 118.95 (d, J_{CF}=27.7 Hz, C47), 118.9 (C19), 118.6 (C42), 110.7 (C25), 71.65 (2 x alkoxy CH_2), 71.6 (2 x alkoxy CH_2), 71.55 (alkoxy CH_2), 71.4 (alkoxy CH_2), 70.7 (C2), 68.2 (C9), 66.4 (d, J_{CF}=7.2 Hz, C34), 66.0 (C32), 64.6 (d, J_{CF}=7.8 Hz, C35), 56.6 (C30), 51.6 (d, *J*=3.8 Hz, C33), 46.0 (C49), 41.2 (C1), 38.8 (C10), 31.4 (C50), 30.5 (C51). ^{19}F NMR (376 MHz, CD_3OD) δ_F ppm -109.4 (48-CF), -121.4 (37-CF). HRMS (ESI) m/z: $[M+H]^+$ calculated for $C_{55}H_{62}{}^{35}Cl_2F_2N_7O_9$: 1072.3954,

found 1072.3921; $C_{55}H_{62}{}^{35}Cl^{37}ClF_2N_7O_9$: 1074.3925, found 1074.3916; $C_{55}H_{62}{}^{37}Cl_2F_2N_7O_9$: 1076.3895, found 1076.3933.

2. Properties Table of **1-4**

Table S1. Physiochemical properties[a] table for compounds **1**-**4**.

Compound	Mwt	cLogP	TPSA (Å^2)	H-bond donors	H-bond acceptors	Rotatable Bonds
**1**	1023.05	9.77	187.47	6	9	27
**2**	966.94	8.48	187.47	6	9	23
**3**	1028.96	6.95	215.16	6	12	27
**4**	1073.02	7.11	224.39	6	13	30

[a]Physiochemical property predictions of **1**-**4** were calculated using SwissADME (swissadme.ch). The cLogP values represent the average of five LogP predictions from the programme.

Figure S1. Screening of 1-4 for HDAC1-HDAC3 degradation.

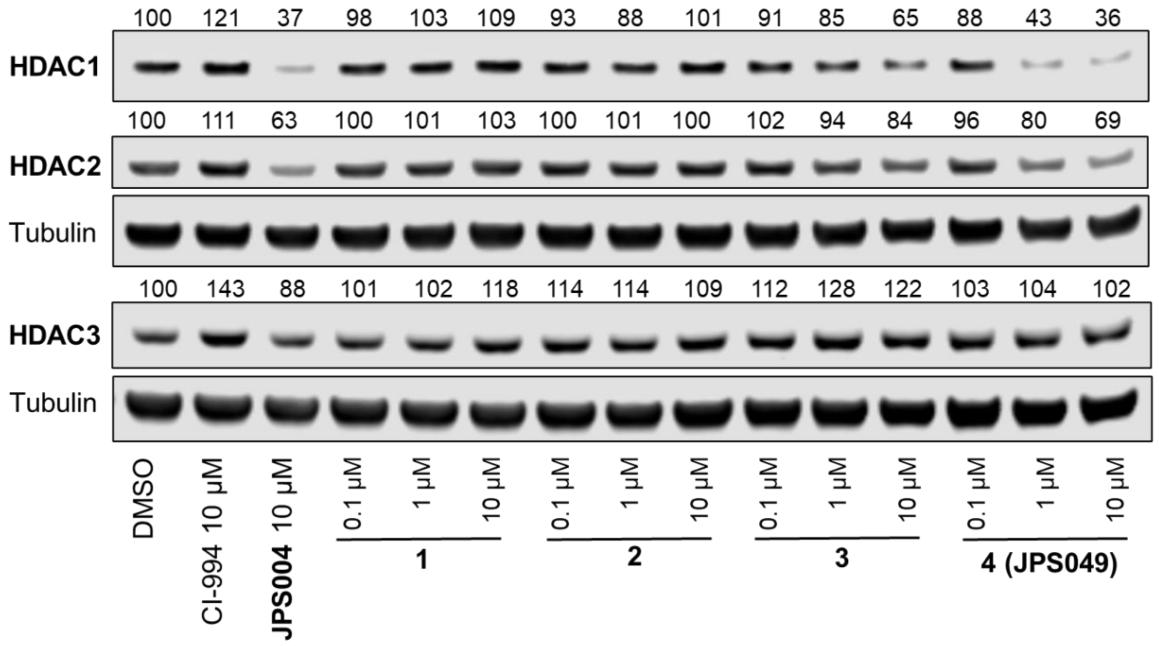

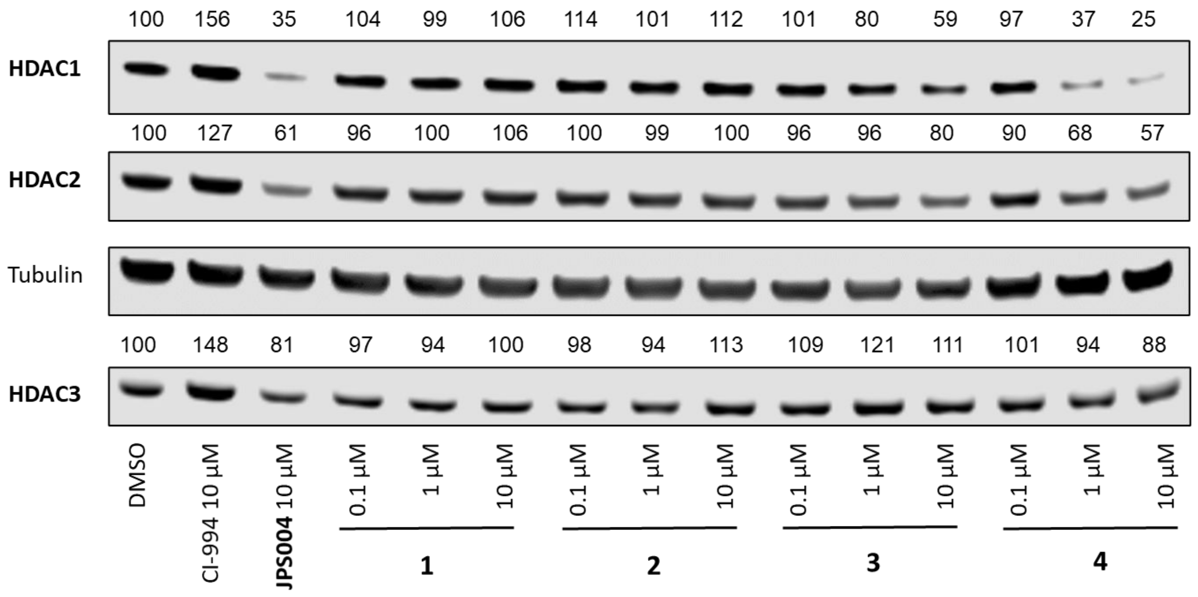

Figure S2. Screening of 1-4 for H3K56ac.

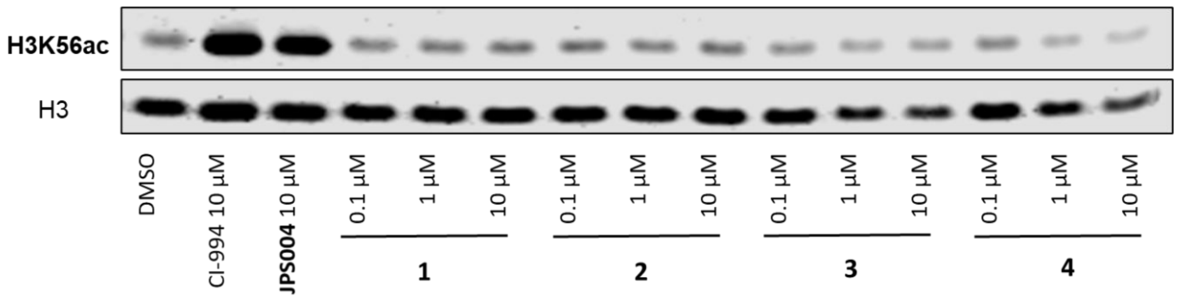

Figure S3. HDAC1, HDAC2 and HDAC3 dose response with compound 4.

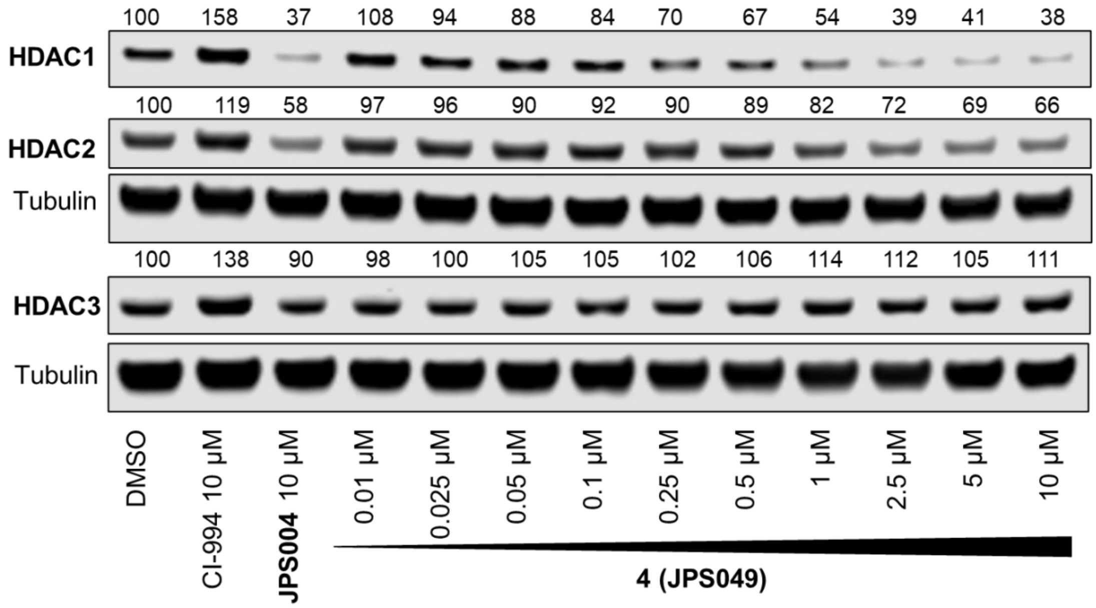

Figure S4. Sin3A and LSD1 dose response with compound 4

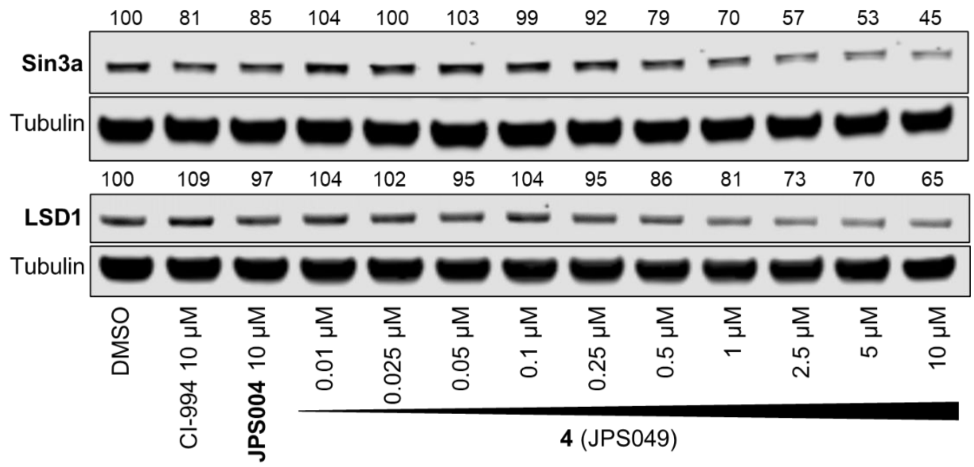

Figure S5. Idasanutlin effects on HDAC1, HDAC2 and HDAC3.

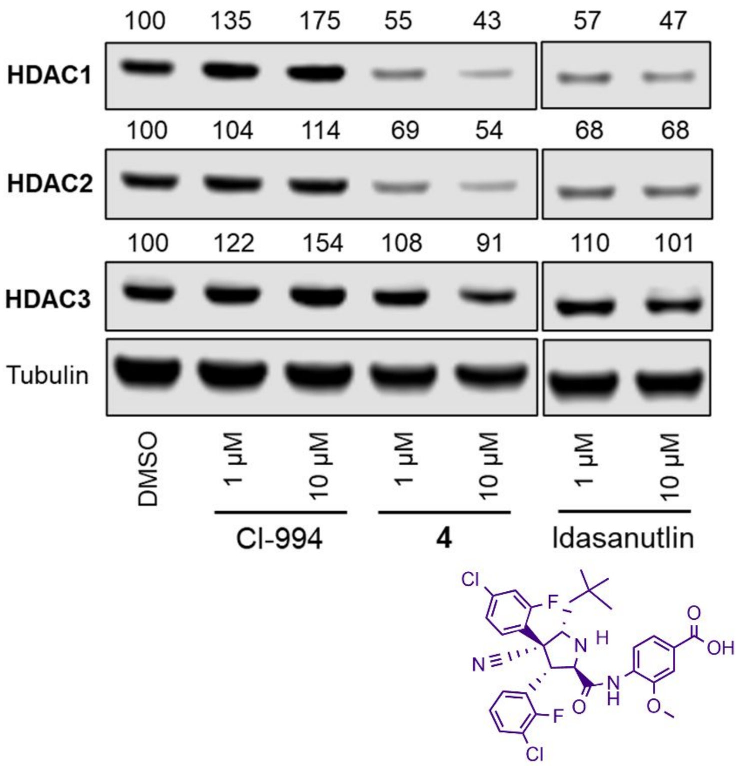

4. NMR spectra for **1-4**

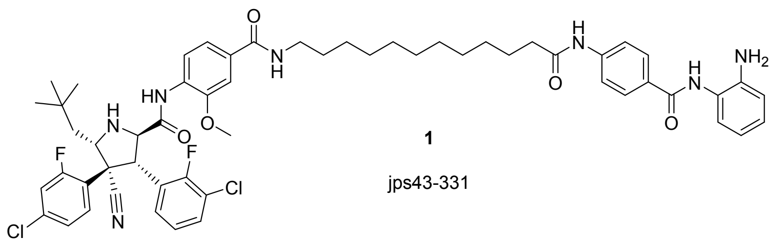

1

jps43-331

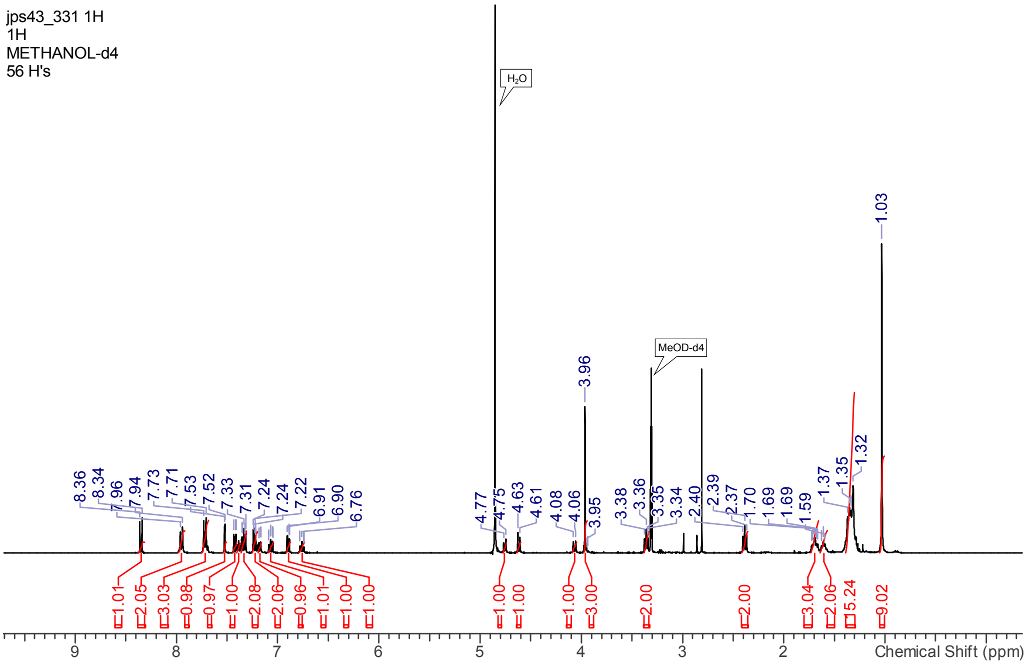

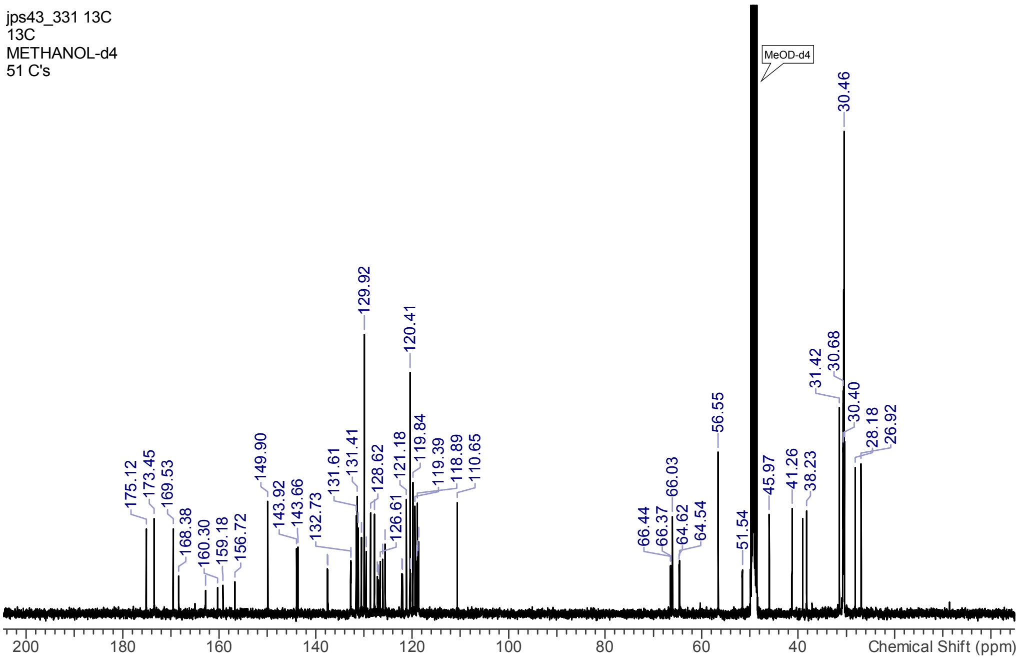

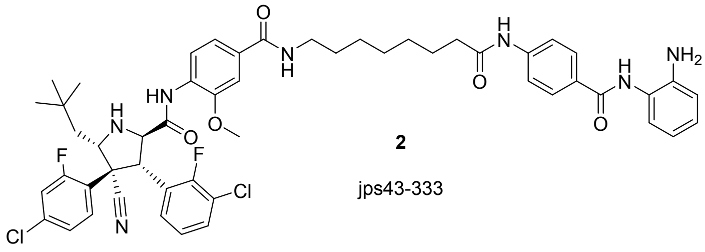

2

jps43-333

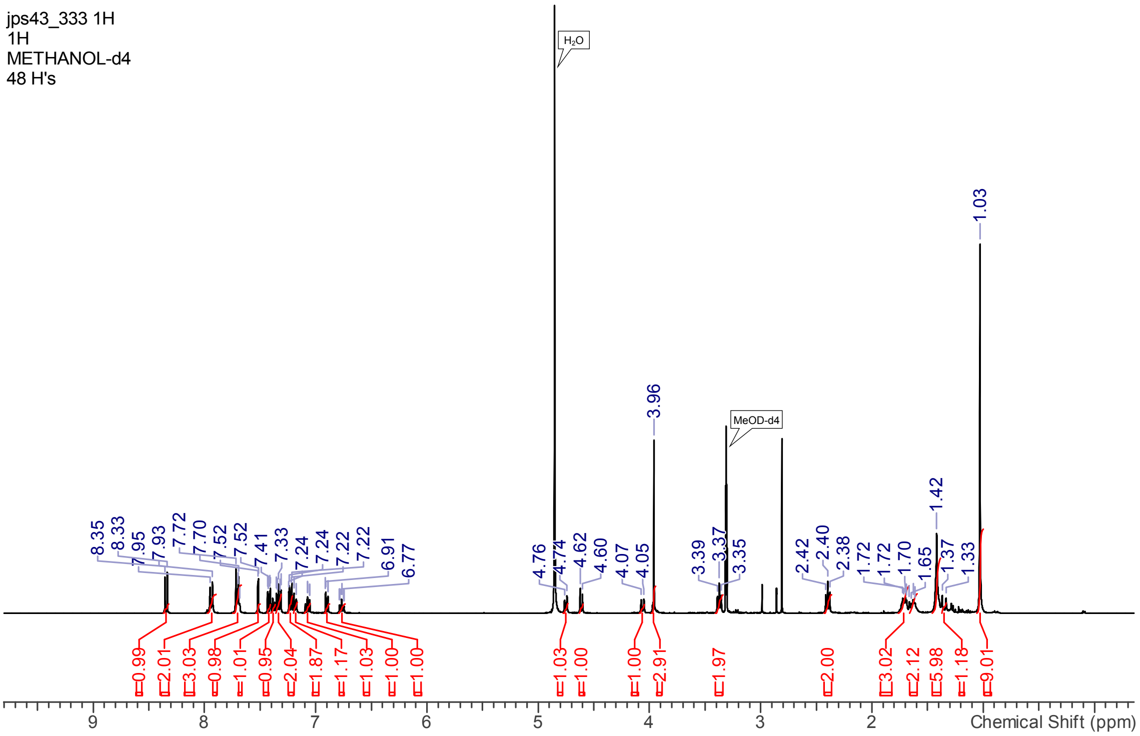
jps43_333 1H
1H
METHANOL-d4
48 H's
H₂O
MeOD-d4
8.35 8.33 7.95 7.93 7.72 7.70 7.52 7.52 7.41 7.33 7.24 7.24 7.22 7.22 6.91 6.77
4.76 4.74 4.62 4.60 4.07 4.05 3.96 3.39 3.37 3.35 2.42 2.40 2.38 1.72 1.72 1.70 1.65 1.42 1.37 1.33 1.03
0.99 2.01 3.03 0.98 1.01 0.95 2.04 1.87 1.17 1.03 1.00 1.00 1.03 1.00 1.00 2.91 1.97 2.00 3.02 2.12 5.98 1.18 9.01
9 8 7 6 5 4 3 2
Chemical Shift (ppm)

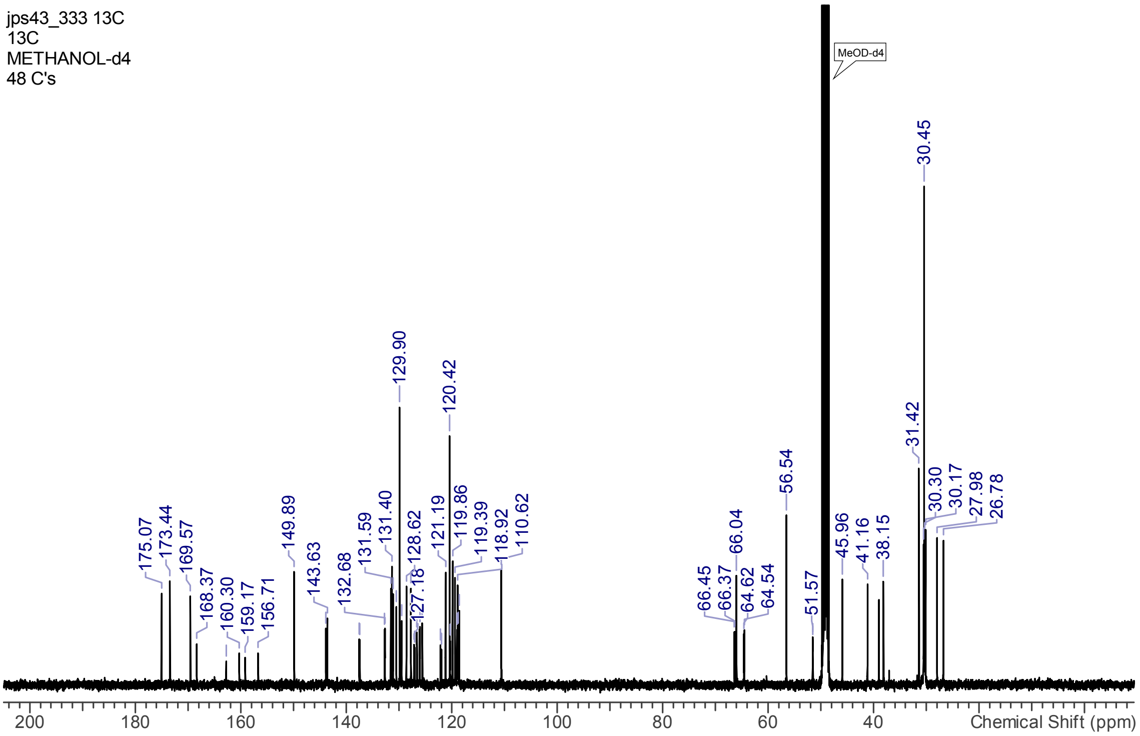
jps43_333 13C
13C
METHANOL-d4
48 C's
MeOD-d4
175.07 173.44 169.57 168.37 160.30 159.17 156.71 149.89 143.63 132.68 131.59 131.40 129.90 128.62 127.18 121.19 120.42 119.86 119.39 118.92 110.62
66.45 66.37 66.04 64.62 64.54 56.54 51.57 45.96 41.16 38.15 31.42 30.45 30.30 30.17 27.98 26.78
200 180 160 140 120 100 80 60 40
Chemical Shift (ppm)

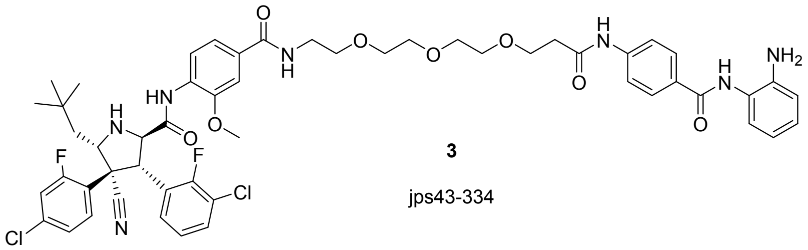
NH2
Cl
F
N

3

jps43-334

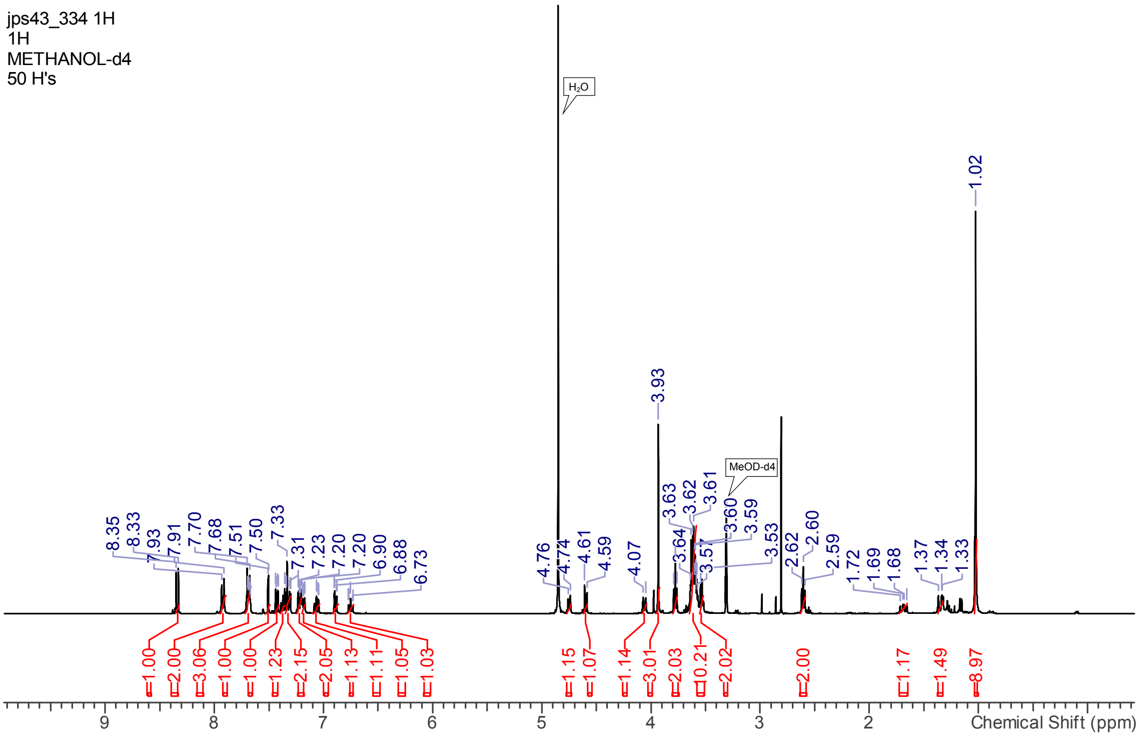
jps43_334 1H
1H
METHANOL-d4
50 H's
H2O
MeOD-d4
Chemical Shift (ppm)

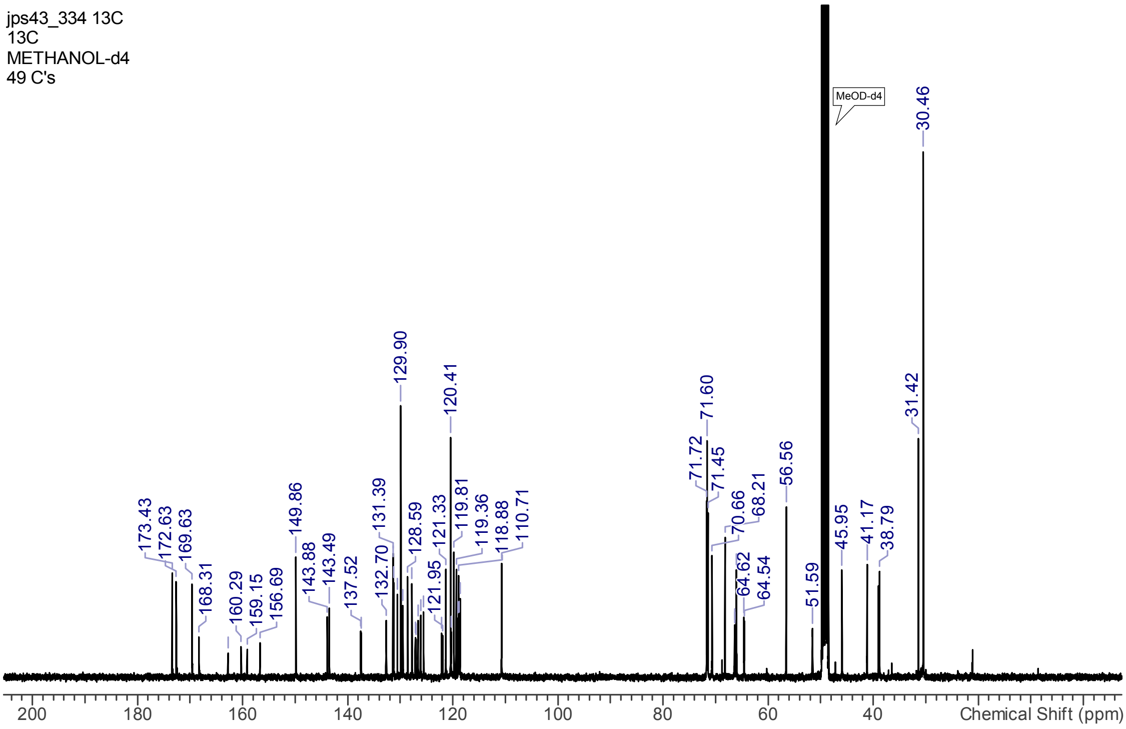
jps43_334 13C
13C
METHANOL-d4
49 C's
MeOD-d4
Chemical Shift (ppm)

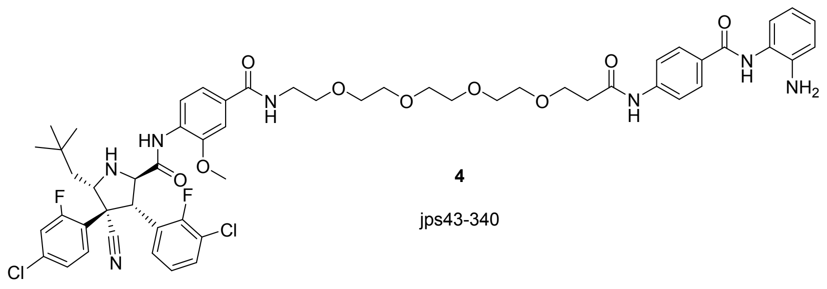

4

jps43-340

jps43_340 1H
1H
METHANOL-d4
54 H's

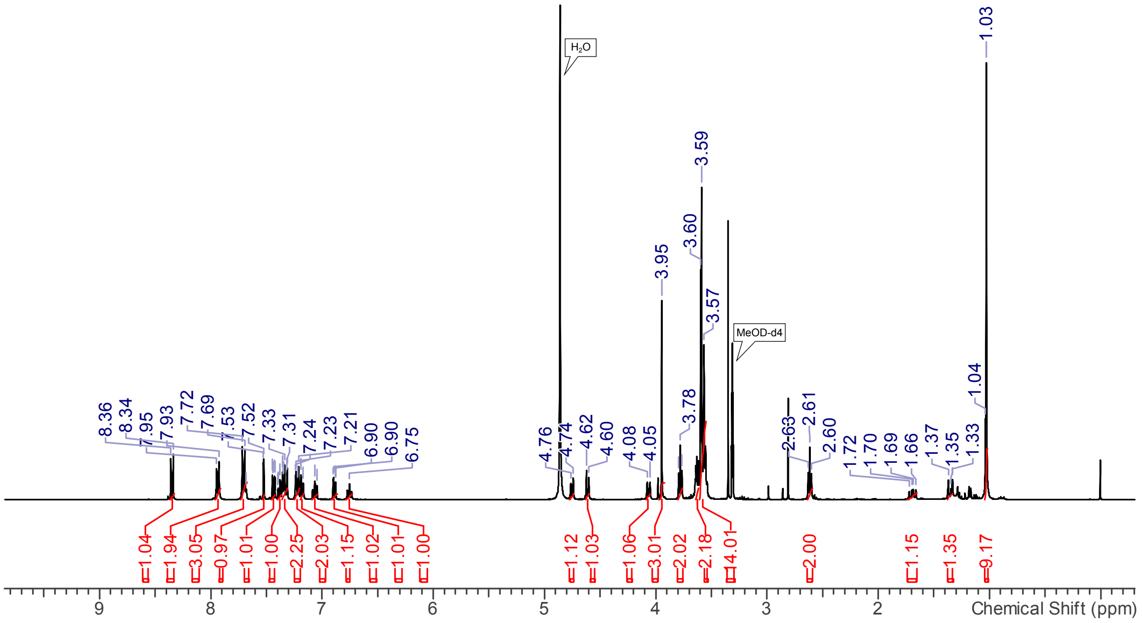

jps43_340 13C
13C
METHANOL-d4
50 C's

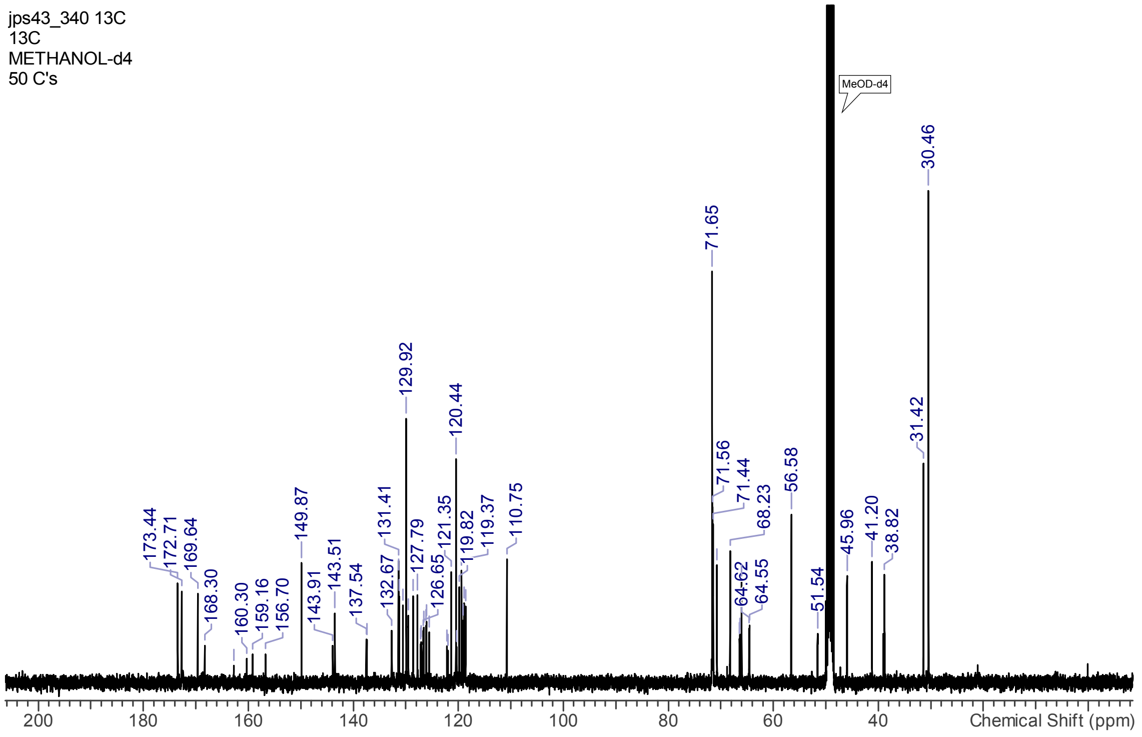

5. UPLC traces for **1-4**

Figure S6. UPLC results for **1** (JPS331), **2** (JPS333), **3** (JPS334) and **4** (JPS340) at 260nm.

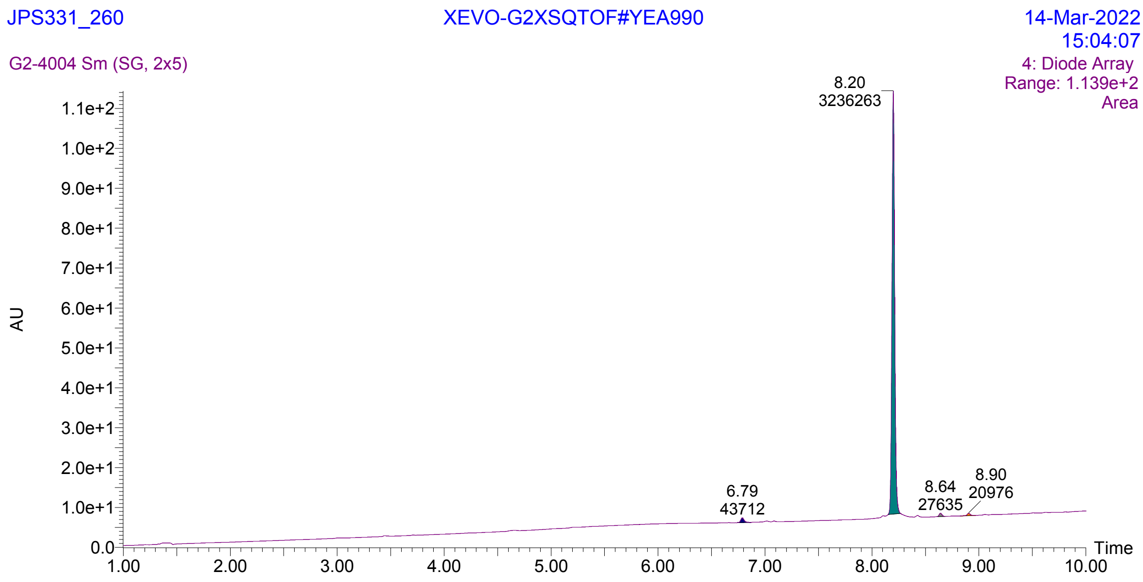

Peak #	Ret. Time (min)	Height (AU)	Area	Ret. Area %
1	6.788	1202169	43711.84	1.313%
2	8.199	105992344	3236263	97.226%
3	8.641	863548	27634.971	0.837%
4	8.904	590069	20975.77	0.630%

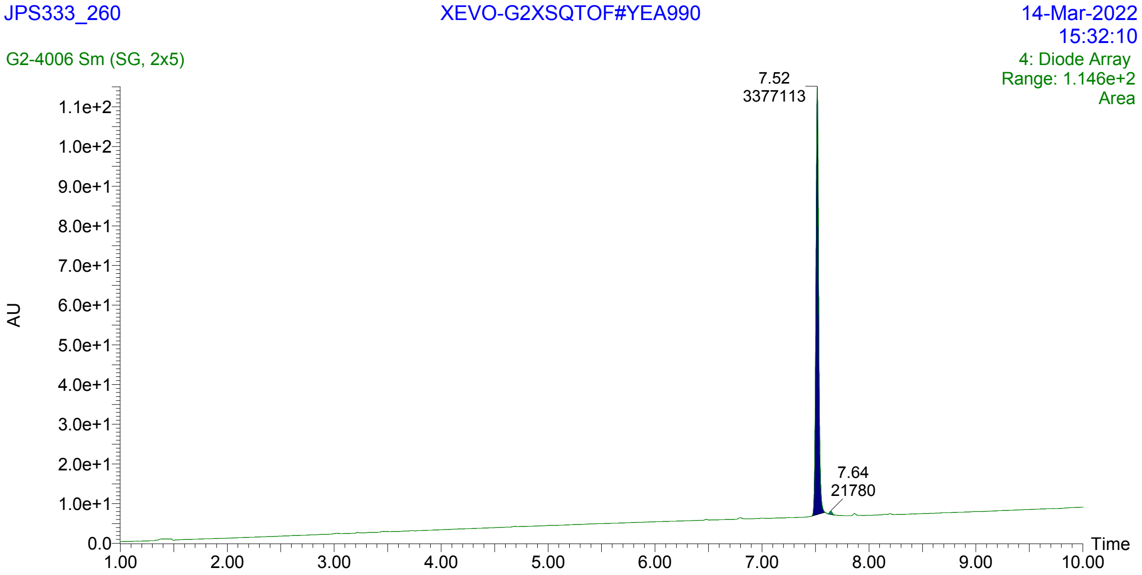

Peak #	Ret. Time (min)	Height (AU)	Area	Ret. Area %
1	7.517	107790080	3377113.25	99.359%
2	7.643	804992	21779.656	0.641%

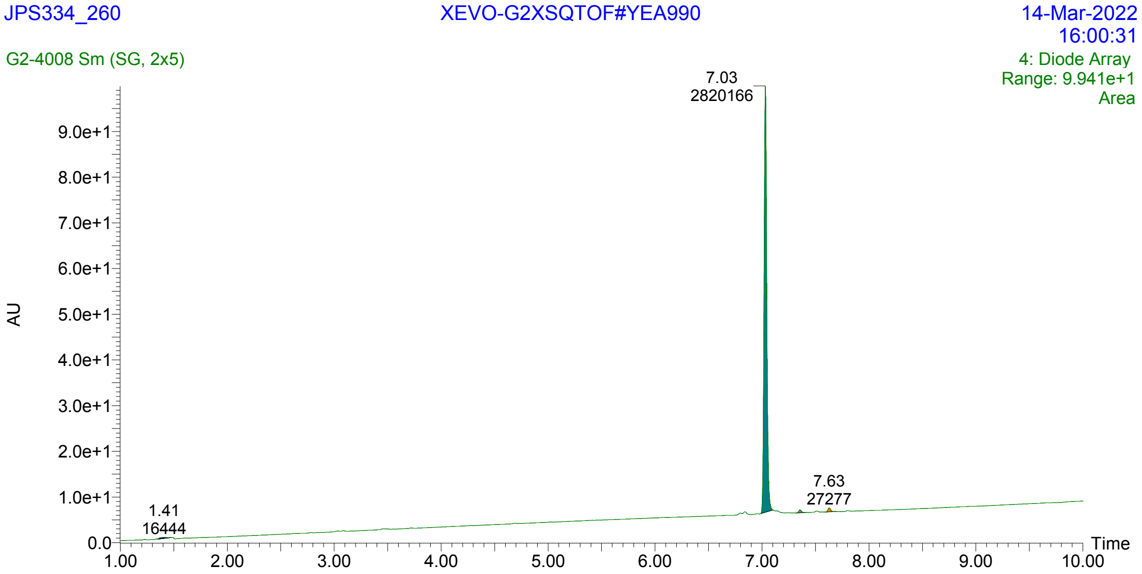

Peak #	Ret. Time (min)	Height (AU)	Area	Ret. Area %
1	7.031	93176072	2820166.25	98.416%
2	7.356	595959	18126.623	0.633%
3	7.628	874743	27277.084	0.952%

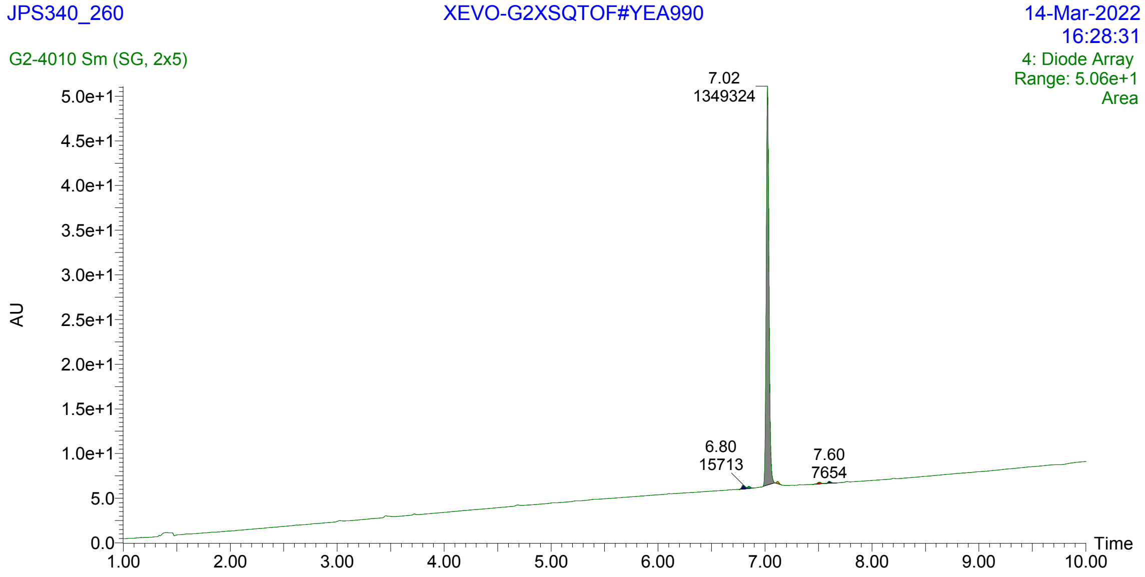

Peak #	Ret. Time (min)	Height (AU)	Area	Ret. Area %
1	6.797	428317	15712.929	1.126%
2	6.851	229807	7622.655	0.546%
3	7.024	44581480	1349323.625	96.693%
4	7.117	254381	6882.45	0.493%
5	7.508	209991	8276.431	0.593%
6	7.602	223526	7653.832	0.548%
